# Genome-Wide Identification and Abiotic Stress Response Analysis of PP2C Gene Family in Woodland and Pineapple Strawberries

**DOI:** 10.3390/ijms24044049

**Published:** 2023-02-17

**Authors:** Lili Guo, Shixiong Lu, Tao Liu, Guojie Nai, Jiaxuan Ren, Huimin Gou, Baihong Chen, Juan Mao

**Affiliations:** College of Horticulture, Gansu Agricultural University, Lanzhou 730070, China

**Keywords:** protein phosphatase 2C, *Fragaria vesca*, *Fragaria ananassa*, genome-wide analysis, expression analysis

## Abstract

Protein phosphatase 2C (PP2C) is a negative regulator of serine/threonine residue protein phosphatase and plays an important role in abscisic acid (ABA) and abiotic-stress-mediated signaling pathways in plants. The genome complexity of woodland strawberry and pineapple strawberry is different due to the difference in chromosome ploidy. This study conducted a genome-wide investigation of the FvPP2C (*Fragaria vesca*) and FaPP2C (*Fragaria ananassa*) gene family. Fifty-six *FvPP2C* genes and 228 *FaPP2C* genes were identified from the woodland strawberry and pineapple strawberry genomes, respectively. *FvPP2Cs* were distributed on seven chromosomes, and *FaPP2Cs* were distributed on 28 chromosomes. The size of the FaPP2C gene family was significantly different from that of the FvPP2C gene family, but both FaPP2Cs and FvPP2Cs were localized in the nucleus, cytoplasm, and chloroplast. Phylogenetic analysis revealed that 56 *FvPP2Cs* and 228 *FaPP2Cs* could be divided into 11 subfamilies. Collinearity analysis showed that both *FvPP2Cs* and *FaPP2Cs* had fragment duplication, and the whole genome duplication was the main cause of *PP2C* gene abundance in pineapple strawberry. *FvPP2Cs* mainly underwent purification selection, and there were both purification selection and positive selection effects in the evolution of *FaPP2Cs*. *Cis*-acting element analysis found that the *PP2C* family genes of woodland and pineapple strawberries mainly contained light responsive elements, hormone responsive elements, defense and stress responsive elements, and growth and development-related elements. The results of quantitative real-time PCR (qRT-PCR) showed that the *FvPP2C* genes showed different expression patterns under ABA, salt, and drought treatment. The expression level of *FvPP2C18* was upregulated after stress treatment, which may play a positive regulatory role in ABA signaling and abiotic stress response mechanisms. This study lays a foundation for further investigation on the function of the PP2C gene family.

## 1. Introduction

Plants are often affected by biotic and abiotic stresses during their growth and development, which have a great impact on crop yield and quality [1]. Plants can respond to stress through various physiological and biochemical reactions such as photosynthesis, glucose metabolism, cell growth and reproduction, and many of their basic regulatory mechanisms involve the reversible phosphorylation of proteins [2,3]. Reversible protein phosphorylation is a protein modification process that is mainly performed by protein kinases (PKs) and protein phosphatases (PPs) [4,5]. PKs can phosphorylate serine (Ser), threonine (Thr), and tyrosine (Tyr), while PPs eliminate phosphate groups for reversal [6]. On the basis of substrate specificity, PPs can be divided into protein serine/threonine phosphatases (STPs), protein tyrosine phosphatases (PTPs), and protein dual specificity phosphatases (DSPTPs). Based on their crystal structure, amino acid sequence, and specific response to inhibitors, PTPs are divided into two types, phosphoprotein metal phosphatases (PPM) and phosphoprotein phosphatases (PPP) [7]. STPs mainly include PP1 and PP2, which are subdivided into PP2A, PP2B, and PP2C due to the subunit structure of PP2 and the requirement for divalent cation activity [8,9,10].

Protein phosphatase 2C (PP2Cs) is a negative regulator of serine/threonine residue protein phosphatase, and the activity of PP2Cs depends on Mg^2+^ or Mn^2+^ [7]. With regard to the PP2C protein structures, its C-terminus has a relatively conserved catalytic domain, and its N-terminus is an extension region that is not highly conserved and has different functions. Meanwhile, this extension region is also unique to PP2C proteins [11]. PP2Cs are the most numerous members of the plant protein phosphokinase family and are involved in multiple signal transduction pathways with different regulatory mechanisms. It has been shown that PP2Cs are evolutionarily conserved in fungi, archaea, bacteria, plants, and animals and participate in different stress-regulated pathways and even play negative regulatory roles [12].

The phytohormone abscisic acid (ABA) signaling pathway is an important pathway for higher plants to regulate biotic and abiotic stress, and plays an important role in regulating plant tolerance and growth and development [13]. This pathway mainly involves the ABA receptor protein (PYR/PYL/RCAR), PP2Cs, and SNF1-associated protein kinase 2 (SnRK2s) [14,15,16]. ABA can reportedly interact with the receptor protein PYR/PYL/RCAR and regulate SnRK2-type protein kinase activity through negative regulation, which plays an important role in regulating PP2C in connection with transduction pathways [12,17].

The PP2Cs gene family has been identified in many plants such as *Triticum aestivum* [18], *Vitis vinifera* [19], *Oryza sativa* [20], *Arachis hypogaea* [21], *Zea mays* [22], *Brachypodium distachyum* [12], *Glycine max* [23], and *Sorghum bicolor* [23], which contain 80, 27, 132, 79, 130, 86, 158, and 53 members, respectively. PP2Cs have different functions in the stress-response pathways of different plants [24,25,26]. In the study of *Arabidopsis thaliana*, 80 *PP2Cs* genes were identified and divided into 13 groups. Among them, six out of nine members of subfamily A had negative regulation effects on ABA, namely, ABI1, ABI2, AHG1, AHG3/ATPP2C-A, HAB1, and HAB2 [27,28,29,30,31,32]. Subfamily B is involved in the mitogen-activated protein kinase (MAPK) signaling pathway, which is inactivated by the phosphorylation of MAPKs [33,34]. Subfamily C is involved in flowering regulation [35]. Subfamily D is involved in response to saline-alkali stress [36]. Subfamily E participates in transpiration by regulating stomatal signaling and subfamily F is involved in the induction of bacterial stress responses [37,38,39]. *ZmPP2C-A10* in maize has been transferred to *A. thaliana*, which proves that it is a negative regulator of drought tolerance [40]. Xue et al. verified the upregulated expression of *ZmPP2C* in subgroup G under salt stress [29]. Singh et al. found that subgroup A of the *ZmPP2C* genes could positively regulate abiotic stress and negatively regulate the ABA signaling pathway in rice [15]. In strawberry, the *FaABI1* gene has a negative regulatory effect on maturation [41]. The *PP2C* gene of subfamily B in alfalfa can respond to stress induction and play a negative regulatory role in the MAPK signaling pathway [42]. In millet, SiPP2CA8 can interact with SiRCAR3, a protein similar to the millet ABA receptor, and participate in the ABA signal transduction pathway [18].

Strawberry has high economic, medicinal, and medical value. Its delicious flesh is loved by people, but is susceptible to drought, low temperature and other external conditions. The diploid woodland strawberry, with its short growth cycle, relatively small genome (about 240 Mb), efficient genetic transformation, and abundant transcriptome data resources, is a model plant for the study of strawberry gene function [43]. Octaploid pineapple strawberry has been a popular cultivar in recent years. Its genetic background is narrow, and its resistance to stress is weak [44]. The genome complexity of woodland strawberry and pineapple strawberry is different due to the difference in the chromosome ploidy. Therefore, this study identified the PP2C gene family based on the genomic screening of woodland and pineapple strawberries, and systematically analyzed them by the bioinformatics method. By comparing the similarities and differences between the two varieties and treating the strawberry test-tube seedlings with simulated stress, the PP2C gene family of strawberry was preliminarily analyzed and the expression patterns of *PP2Cs* in strawberry under various stress conditions were explored. This study provides a basis for the study and utilization of the PP2C gene function in strawberry.

## 2. Results

### 2.1. Identification of PP2C Gene Family in Woodland and Pineapple Strawberries

A total of 56 and 228 *PP2C* genes were identified in woodland strawberry and pineapple strawberry by homologous screening, respectively. These are named *FvPP2C01*-*FvPP2C56* and *FaPP2C01*-*FaPP2C228,* according to their Latin abbreviation. Physicochemical characteristic analysis showed that the length of amino acids encoded by *FvPP2C* genes varied from 271 aa to 1080 aa, among which *FvPP2C01* was the longest and *FvPP2C25* was the shortest. Most genes contained around 380 amino acids. The molecular weight ranged from 30,328.15 to 119,808.08 Da, and the pI ranged from 4.34 to 9.30, of which acid protein accounted for 76.79% (Supplementary Table S1). Compared with FvPP2C, the gene length, amino acid length, and molecular weight of FaPP2C were significantly different, while the isoelectric point and protein hydrophilicity were not significantly different. The lengths of the FaPP2C proteins varied from 87 (FaPP2C221) to 2106 (FaPP2C36) and molecular weight varied from 9255.33 Da (FaPP2C221) to 236,904.86 Da (FaPP2C36). Most of the FaPP2C proteins were acidic, but the D and E subfamilies were mainly alkaline (Supplementary Table S2). In addition, except for FaPP2C113, FaPP2C152 and FaPP2C179, all of the other FaPP2Cs and FvPP2Cs were hydrophilic proteins (Supplementary Tables S1 and S2).

The subcellular localization prediction showed that FvPP2C and FaPP2C were mainly localized in three organelles: nucleus, cytoplasm, and chloroplast. Furthermore, FvPP2C49 was present in the cytoplasmic matrix. In pineapple strawberry, only FaPP2C132 might be located in the Golgi apparatus, FaPP2C211 in the cytoskeleton, and FaPP2C211 in the endoplasmic reticulum (Supplementary Tables S1 and S2).

### 2.2. Phylogenetic Tree Analysis

**Figure 1:**
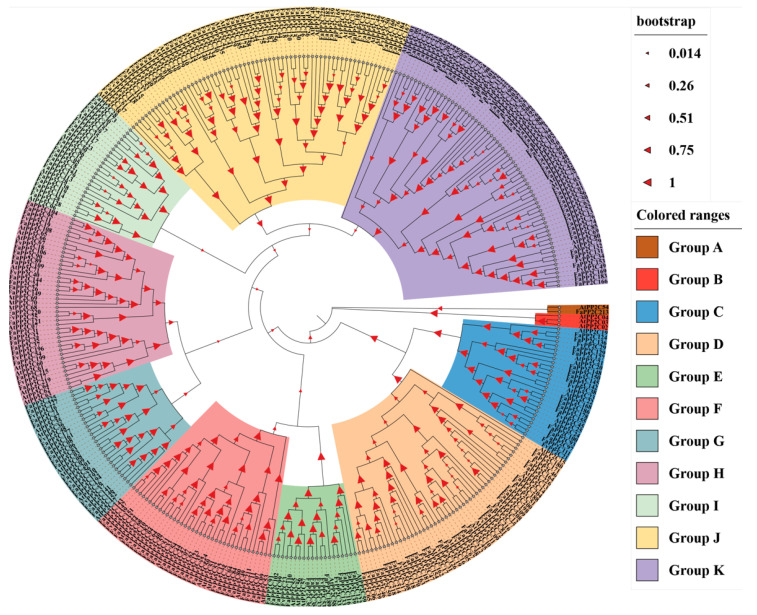
Phylogenetic analysis of the PP2C gene family from *F. vesca*, *F. ananassa*, and *A. thaliana*. Note: Phylogenetic trees were constructed using the PP2C protein sequences. Maximum parsimony was adopted, and the bootstrap value was set to be equal to 1000.

Phylogenetic trees were constructed using the PP2C protein sequences of *A. thaliana*, *F. vesca*, and *F. ananassa*, and the phylogenetic relationship between strawberry and *A. thaliana* was identified (Figure 1). The results showed that 56 FvPP2Cs and 228 FaPP2Cs were clearly divided into 11 subfamilies (A–K). Subgroup B only contained PP2C proteins from *A. thaliana*. Subgroups J and K contained the most PP2C members, among which subgroup K included nine FvPP2Cs and 46 FaPP2C proteins, and subgroup J included 10 FvPP2Cs and 42 FaPP2C proteins. In addition, PP2C proteins of the same species tend to form independent branches in the same subfamily, that is, woodland strawberry proteins clustered together and pineapple strawberry proteins clustered together.

### 2.3. Gene Structure Analysis and Conserved Motif Analysis

To further understand the phylogeny and function of the PP2C gene family in strawberry, the *FvPP2Cs* and *FaPP2Cs* gene structures and protein conserved motifs were analyzed. Most genes in the same subfamily had the same number of exons and similar exon lengths. In woodland strawberry (Supplementary Figure S1A), the number of exons of *FvPP2Cs* varied from 1 to 15. Among them, *FvPP2C11* and *FvPP2C36* were intron deletion types, and *FvPP2C18* and *FvPP2C20* lacked upstream and downstream structures. Different intron lengths may affect gene functions. *FvPP2C20* in subfamily K had an intron region of more than 6 kb, which was the longest gene in this subfamily. In pineapple strawberry (Supplementary Figure S2A), the number of exons in *FaPP2Cs* ranged from 1 to 35, with relatively few exons in subgroups C, F, and K, ranging from 2 to 5. In addition, *FaPP2C07*, *09*, *20*, *21*, *23*, *27*, and *FaPP2C227* contained only one exon, and there were 12 *FaPP2C* members that did not have upstream and downstream structures. Different gene structures may affect the gene expression and corresponding protein function.

Using MEME motif search tool, eight motifs were identified for FvPP2Cs and FaPP2Cs, respectively. The number of conserved motifs in the FvPP2C proteins varied from three to seven, but the composition and distribution of conserved motifs within the same subfamily were similar. Both FvPP2C13 and FvPP2C25 contained only three motifs, namely, motifs 1, 2, 7, and motifs 2, 4, 8. Subgroups C and D contained motif 6 at the N-terminus and the remaining subgroups contained motif 7 at the N-terminus (Supplementary Figure S1B). In pineapple strawberry, most FaPP2C proteins contained one to six motifs. Among them, FaPP2C02, 13, 125, 192, 194, and FaPP2C210 only contained motif 1, FaPP2C221 only contained motif 5, and FaPP2C225 only contained motif 2. The same as FaPP2Cs, subgroups C and D contained motif 6 at the N-terminus, but the remaining subgroups contained motif 1 at the N-terminus (Supplementary Figure S2B). It is worth noting that the amino acid sequence of motif 4 was the same by comparing eight motifs between the two species (Supplementary Figure S3). It is speculated that motif 4 plays an important role in strawberry.

### 2.4. Chromosomal Location and Collinearity Analysis

**Figure 2:**
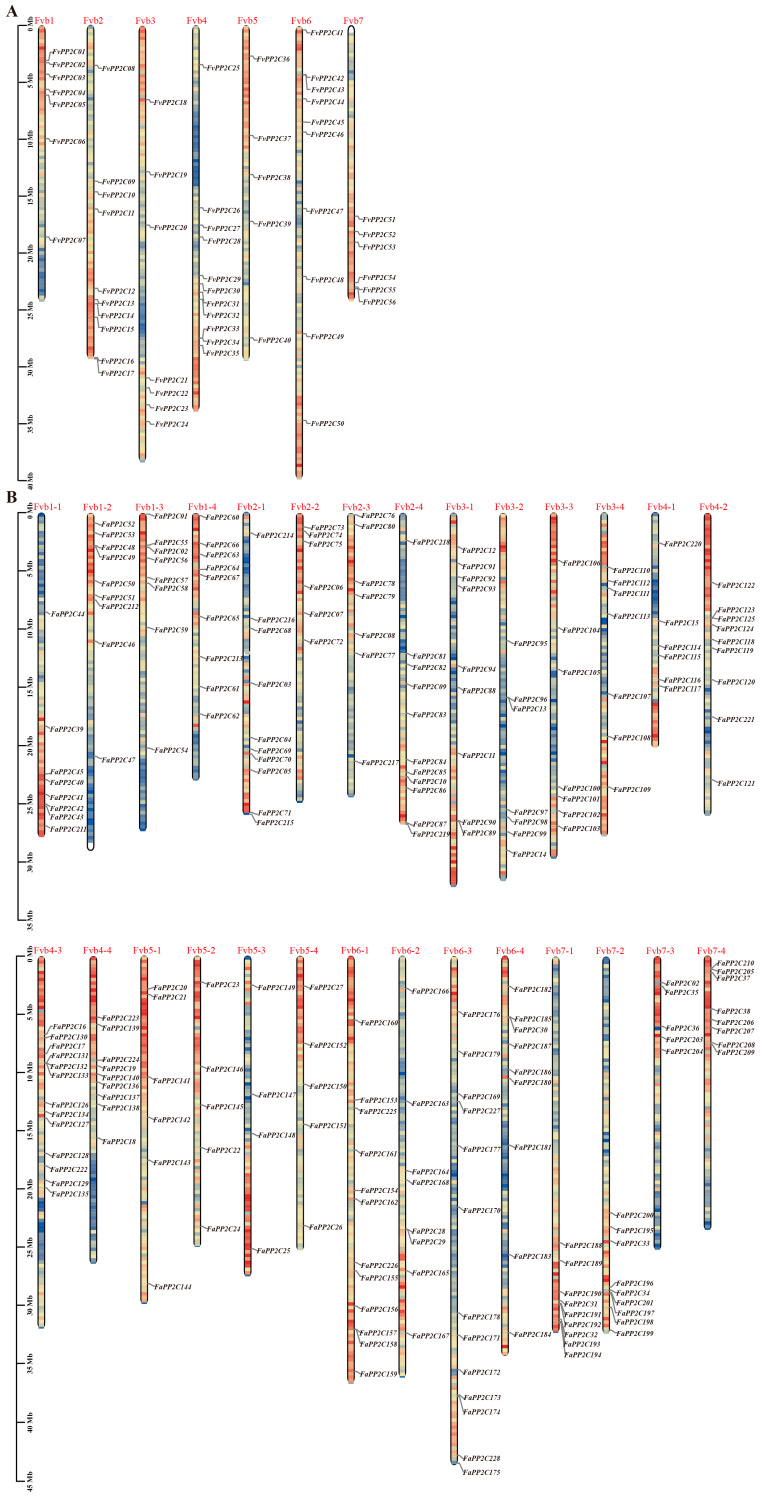
Chromosome distribution of the PP2C gene family in woodland strawberry and pineapple strawberry. Note: The left scale indicates the chromosome length (Mb), with PP2C gene markers on the right side of each chromosome. Different chromosomal colors indicate different gene densities, with red indicating the highest density and blue the lowest density. (**A**) Chromosome distribution of *FvPP2Cs*. (**B**) Chromosome distribution of *FaPP2Cs*.

The location of *FvPP2Cs* (Figure 2A) and *FaPP2Cs* (Figure 2B) on the strawberry chromosome were analyzed. The results showed that 56 *FvPP2Cs* were unevenly distributed on seven chromosomes (Fvb1-Fvb7). For them, Fvb4 was the most distributed (11), followed by Fvb2 and 6, and Fvb5 was the least distributed (5). Overall analysis showed that *FvPP2C* genes on Fvb1 and 6 were concentrated in the first half of the chromosome, while *FvPP2C* genes on Fvb2, 3, 4, and 7 were mainly concentrated in the second half of the chromosome. Two pairs of genes, *FvPP2C01* and *FvPP2C02* and *FvPP2C55* and *FvPP2C56*, were close to each other on the chromosomes, respectively. The 228 *FaPP2C* genes were unevenly mapped on 28 chromosomes (Fvb1-1 to Fvb7-4), ranging from 4 (Fvb5-3) to 13 (Fvb4-3 and Fvb6-3), and always existed in the regions with high gene density. The genes distributed on Fvb2-2, Fvb7-3, and Fvb7-4 were all in the upper part of the chromosome, while Fvb7-1 and Fvb7-2 showed the opposite.

**Figure 3:**
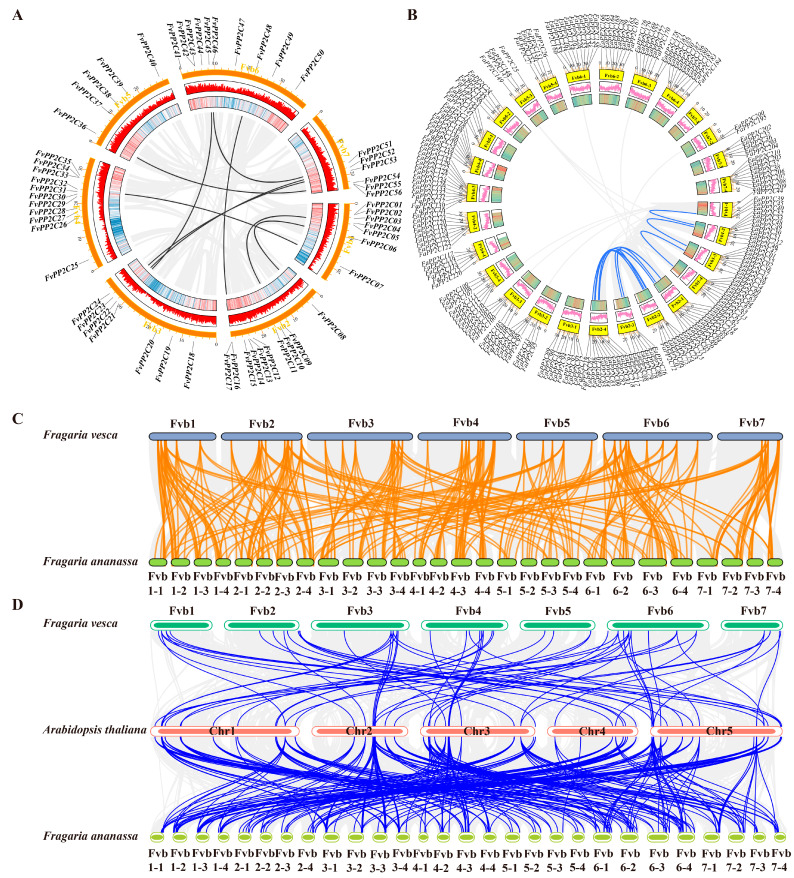
Collinearity analysis of the PP2C gene family. Note: (**A**) Collinearity analysis of *FvPP2Cs*. The orange bars represent chromosomes. The gray lines represent all collinear genes in the woodland strawberry, and black lines represent the *FvPP2C* collinearity genes. (**B**) Collinearity analysis of *FaPP2Cs*. The yellow bars represent chromosomes. The gray lines represent all collinear genes in the pineapple strawberry, and blue lines represent the *FaPP2C* collinearity genes. (**C**) Collinearity analysis of the *PP2C* genes between woodland strawberry and pineapple strawberry. The gray lines denote collinearity between all genes and the orange lines denote collinearity between the two PP2C family members. (**D**) Collinearity analysis of the *PP2C* genes in strawberry to *A. thaliana*. The gray lines represent collinearity between all genes, and the blue lines represent collinearity of the *PP2C* genes between woodland strawberry and *A. thaliana* or between pineapple strawberry and *A. thaliana*.

To further understand the amplification mechanism of *FvPP2Cs* and *FaPP2Cs*, we investigated the collinearity of *PP2Cs* in the woodland strawberry and pineapple strawberry genomes (Figure 3A,B). The results showed that there were nine pairs of collinearity genes in the FvPP2C gene family and 11 pairs of collinearity genes in the FaPP2C gene family, all of which were fragment duplicates. Interestingly, each gene pair belonged to the same subfamily with high homology and their gene structure and conserved motifs were very similar. Unlike *FvPP2C*, the collinear genes of *FaPP2C* clustered on Fvb1-1 to Fvb2-4. At the same time, through the analysis of collinearity between woodland strawberry and pineapple strawberry, we speculated that the whole genome duplication was the main reason for the abundance of *PP2C* genes in the pineapple strawberry (Figure 3C). In addition, we also carried out the collinearity analysis between strawberry and *A. thaliana*, and found that the collinearity gene pairs between pineapple strawberry and *A. thaliana* were significantly more than those between woodland strawberry and *A. thaliana* (Figure 3D).

### 2.5. Evolutionary Selection Pressure and Codon Usage Bias Analysis

**Figure 4:**
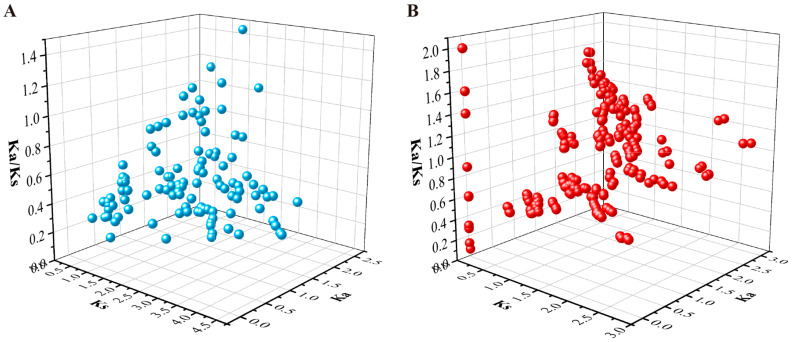
Evolutionary selection pressure analysis of the *PP2C* homologous gene pairs. Note: The X axis represents the Ka value, the Y axis represents the Ks value, and the Z axis represents the ratio of Ka to Ks. (**A**) Evolutionary selection pressure analysis of the *FvPP2C* homologous genes. (**B**) Evolutionary selection pressure analysis of the *FaPP2C* homologous genes.

The Ka/Ks ratio can be used to determine whether there is selection pressure acting on the protein-coding genes, which plays an important role in the evolutionary analysis of gene families. Ks means synonymous substitution, which does not affect amino acid species. Ka denotes a non-synonymous substitution that alters the expression of amino acids [45]. The Ka value, Ks value, and Ka/Ks ratio were analyzed for the homologous gene pairs among *FvPP2Cs* (Figure 4A) and *FaPP2Cs* (Figure 4B). The Ka/Ks value of most *FvPP2C* gene pairs was less than 1, but the number of homologous gene pairs with a Ka/Ks ratio greater than 1 and less than 1 tended to be consistent in pineapple strawberry. These data indicate that these *FvPP2C* were primarily under purifying selection during evolution, while both positive and purifying selection existed in pineapple strawberry.

**Figure 5:**
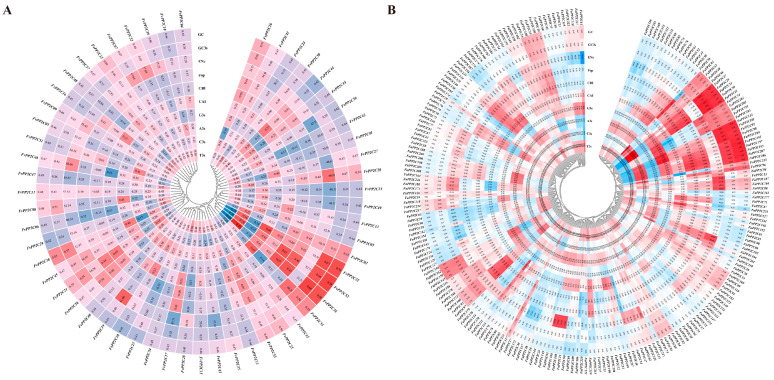
Codon parameter analysis of the *PP2C* genes in strawberry. Note: “A3s, G3s, C3s, and T3s” refer to the synonymous codon corresponding base frequency on the third; “CAI” refers to the codon adaptation index; “CBI” refers to the codon bias index; “FOP” refers to the frequency of optimal codons; “ENc” refers to the effective number of codon; “GC3s” refers to the amount of the third codon (G+C); “GC” refers to the count of genes (G+C). (**A**) Codon parameter analysis of *FvPP2Cs*. (**B**) Codon parameter analysis of *FaPP2Cs*.

**Figure 6:**
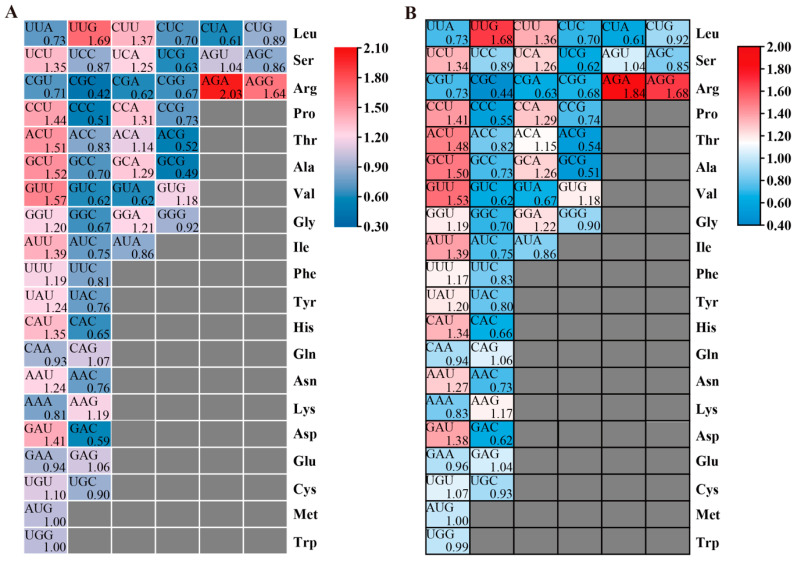
The relative synonymous codon usage (RSCU) analysis of the PP2C gene family in woodland strawberry and pineapple strawberry. Note: A color gradient mapped from low (blue) to high (red) indicates an increase in RSCU. (**A**) The RSCU analysis of the FvPP2C gene family. (**B**) The RSCU analysis of the FaPP2C gene family.

Codon bias analysis is helpful to study species evolution and environmental adaptability. The ENc and CAI of the *FvPP2C* and *FaPP2C* gene family were analyzed. In woodland strawberry, the minimum ENc value of *FvPP2C* was 47.71 (*FaPP2C17*), and the maximum ENc value was 60.20 (*FvPP2C55*). The CAI value ranged from 0.16 to 0.23 (Figure 5). In pineapple strawberry, the ENc value of *FaPP2C* ranged from 40.68 to 61, and the CAI value ranged from 0.14 to 0.24 (Figure 5). Therefore, it was speculated that the codon bias of *FvPP2C* and *FaPP2C* was weak. However, both *FvPP2C* and *FaPP2C* genes in subgroup K showed relatively strong codon preference overall, favoring codons with G/C endings and high G/C content. The relative synonymous codon usage (RSCU) analysis results of the FvPP2C and FaPP2C gene family members were obtained by averaging the corresponding codon RSCU values of amino acids (Figure 6). As can be seen from Figure 6, *FaPP2C* and *FvPP2C* showed certain similarities. The strongest codon preference was found for AGA.

### 2.6. Cis-Acting Elements Analysis

**Figure 7:**
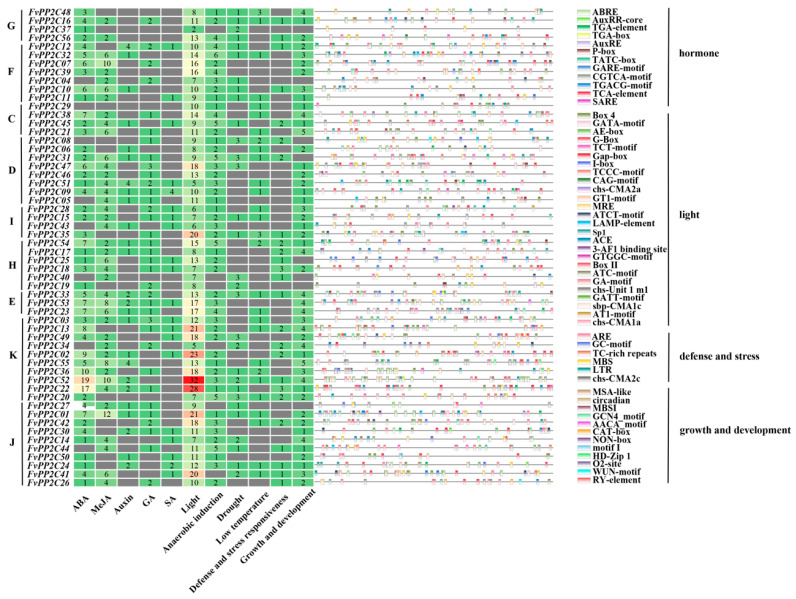
*Cis*-regulatory element analysis of the *FvPP2C* genes. Note: Different letters on the left indicate different subgroups (C–K). Different colors on the right represent different elements. All elements can be divided into four categories, namely, hormone response elements, light response elements, defense and stress response elements, and growth and development related elements. The total number of abscisic acid (ABA), methyl jasmonate (MeJA), auxin, gibberellin (GA) and salicylic acid (SA) response elements, light response elements, anaerobic induction elements, drought response elements, low temperature response elements, defense and stress response elements, and growth and development response elements are shown in the heat map.

**Figure 8:**
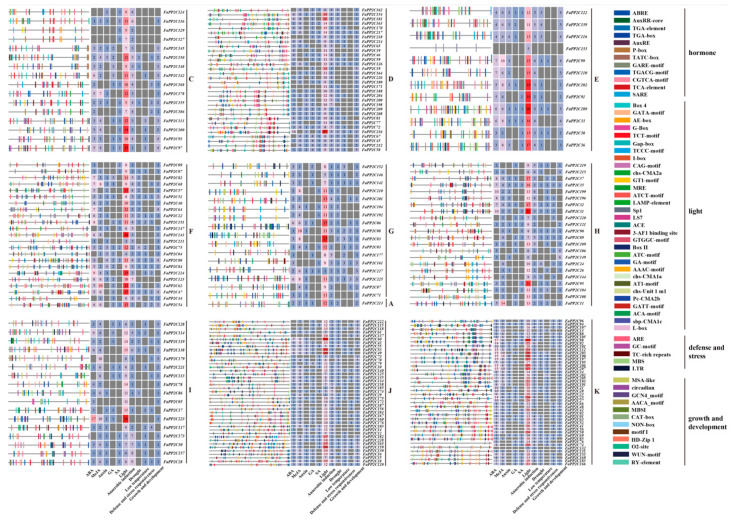
*Cis*-regulatory element analysis of the *FaPP2C* genes. Note: Based on different subfamilies (C–K), the analysis of *cis*-elements was carried out. Different colors on the right represent different elements. All elements can be divided into four categories, namely, hormone response elements, light response elements, defense and stress response elements, and growth and development related elements. The total number of hormone response elements (ABA, MeJA, auxin, JA, SA), light response elements, anaerobic induction elements, drought response elements, low temperature response elements, defense and stress response elements, and growth and development response elements are shown in the heat map.

To further clarify the potential function of the FvPP2C and FaPP2C gene family, cis-acting elements in the upstream 2 kb region were analyzed. The results showed that both *FvPP2Cs* (Figure 7) and *FaPP2Cs* (Figure 8) contained four types of cis-acting elements including light responsive elements, hormone responsive elements, defense and stress responsive elements, and growth and development related elements. Light responsive elements were present in all *FvPP2Cs* and *FaPP2Cs*, and they accounted for a large proportion. It was speculated that *PP2Cs* was closely related to the light regulation of strawberry. Hormone responsive elements included auxin responsive elements, abscisic acid responsive elements, salicylic acid responsive elements, gibberellin responsive elements, and methyl jasmonate responsive elements. In woodland strawberry, except for *FvPP2C04*, *FvPP2C05*, *FvPP2C08*, *FvPP2C29*, *FvPP2C34*, *FvPP2C40*, *FvPP2C43*, and *FvPP2C44*, the rest of the genes contained ABA responsive elements. Among them, *FvPP2C22* and *FvPP2C52* had the most, with 17 and 19, respectively. Most *FvPP2C* genes contained MeJA responsive elements, with the most in *FvPP2C07* and *FvPP2C52*. In pineapple strawberry, the FaPP2Cs in subgroup K contained more ABA and MeJA responsive elements. There were 17 ABA responsive elements in *FaPP2C98* and 16 MeJA responsive elements in *FaPP2C190*. However, there were relatively few auxin responsive elements, gibberellin responsive elements, and salicylic acid responsive elements in the woodland and pineapple strawberries. Defense and stress response elements included low temperature, drought, anaerobic induction, and defense and stress responsive elements. Most of the *FvPP2Cs* and *FaPP2Cs* contained anaerobic induction elements. It suggests that *PP2Cs* played an important role in the regulation of anaerobic induction. In conclusion, the FvPP2C and FaPP2C gene family can not only perform normal transcriptional activities, but also participate in the light response, hormone response, stress response, and growth and development.

### 2.7. Expression Analysis of FvPP2C Genes by qRT-PCR

**Figure 9:**
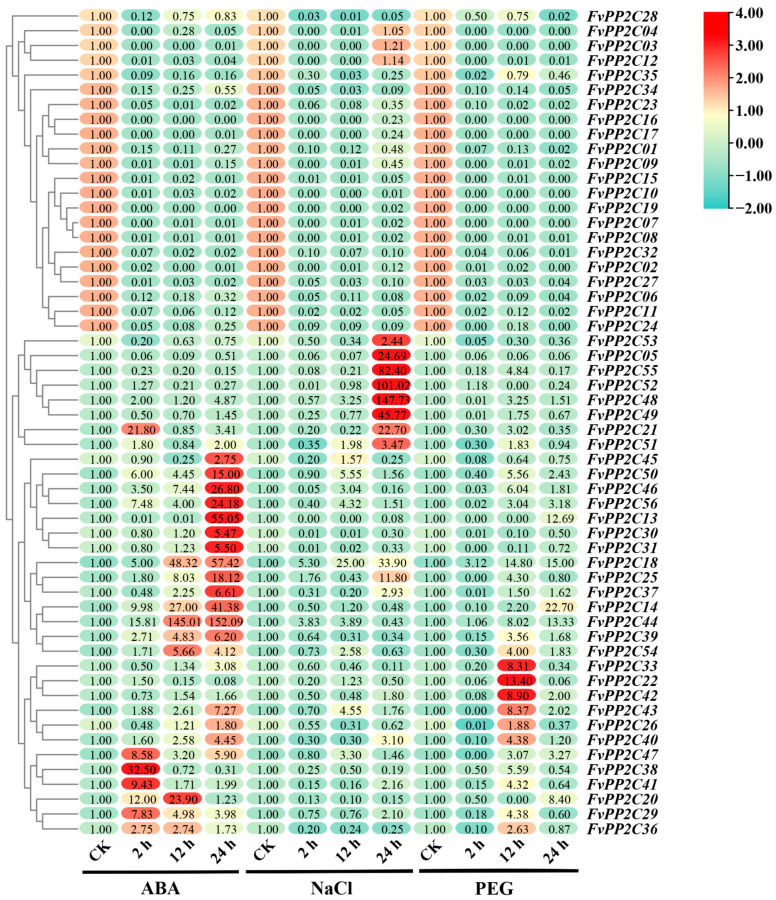
Quantitative real-time PCR analysis of the *FvPP2C* genes in response to the ABA, NaCl, and PEG treatments. Note: The tissue culture seedlings of *F. vesca* were sampled after 2 h, 12 h, and 24 h under 10% PEG, 100 μM ABA, 200 mM NaCl, and equal volume water treated using *FvGAPDH* as the endogenous control. The 2^−∆∆Ct^ method was used to calculate the relative expression. Green represents low expression, yellow represents medium expression, and red represents high expression.

To further understand the role of *PP2Cs* in strawberry stress, the woodland strawberry test-tube seedlings were treated with ABA, NaCl, and PEG, and then the relative expression levels of *FvPP2Cs* in the plant leaves after treatment for 2 h, 12 h, and 24 h were analyzed (Figure 9). Under ABA treatment (concentration = 100 μM), the relative expression of 17 *FvPP2Cs* were upregulated at 2 h, 12 h, and 24 h. Among them, the relative expression levels of *FvPP2C14*, *FvPP2C18*, *FvPP2C25*, *FvPP2C39*, *FvPP2C40*, *FvPP2C43*, *FvPP2C44*, *FvPP2C46*, *FvPP2C50*, and *FvPP2C56* reached the maximum at 24 h. The relative expression level of *FvPP2C44* was the highest, about 152 times that of the control. In addition, the relative expression levels of *FvPP2C21*, *FvPP2C29*, *FvPP2C36*, *FvPP2C38*, *FvPP2C41*, and *FvPP2C47* were significantly upregulated at 2 h, and *FvPP2C21* and *FvPP2C38* were 21 and 32 times that of those in the control group, respectively. The relative expression levels of *FvPP2C14*, *FvPP2C18*, *FvPP2C20,* and *FvPP2C44* were also expressed at higher levels at 12 h. After NaCl treatment (concentration = 200 mM), only the relative expression levels of *FvPP2C18* were upregulated within 2 h, 12 h, and 24 h, with 5.3% upregulated at 2 h, 25.0% upregulated at 12 h, and 33.9% upregulated at 24 h. Furthermore, *FvPP2C05*, *FvPP2C21*, *FvPP2C25*, *FvPP2C48*, *FvPP2C49*, *FvPP2C52,* and *FvPP2C55* showed the most significant changes at 24 h (i.e., 24.69-, 22.7-, 11.8-, 147.73-, 45.77-, 101.02-, and 82.4-fold) of the control. Under 10% PEG6000 treatment, only the relative expression levels of *FvPP2C18* and *FvPP2C44* were upregulated at 2 h, 12 h, and 24 h. Only two genes were upregulated at 2 h, while most genes were upregulated at 12 h and downregulated at 24 h. In general, the *FvPP2C* genes were downregulated under PEG treatment, but the range of changes was not obvious.

## 3. Discussion

PP2Cs belongs to an important group of protein phosphatases and is the largest protein phosphatase family in plants. They participate in the stress-signaling pathway of plants through the dephosphorylation and phosphorylation reactions of the substrate proteins [46]. In *A. thaliana*, *PP2C* genes are reportedly induced by ABA at the transcription level, and PP2C-enzyme-catalyzed reversible phosphorylation is the mechanism of negative regulation in the ABA signaling pathway [47,48]. Moreover, the *PP2C* gene is conserved in the process of evolution [34]. In this study, we comprehensively analyzed the *FvPP2C* gene in woodland strawberry and *FaPP2C* in pineapple strawberry including genome-wide identification, phylogenetic relationships, gene structures, conserved motifs, chromosomal location, collinear relationships, evolutionary selection pressure, codon usage bias, *cis*-acting elements, and expression patterns. Fifty-six *FvPP2C* and 228 *FaPP2C* genes were identified by homology comparison (Supplementary Tables S1 and S2). Obviously, the amount of *FvPP2C* in woodland strawberry was much less than that in *A. thaliana* (80) [29], rice (78) [29], wheat (95) [49], apple (128) [50], and peach (74) [50], while the amount of *FaPP2C* in pineapple strawberry was higher. In addition, 56 *CsPP2Cs* were identified in cucumber, which was consistent with woodland strawberry. The results showed that the FvPP2C gene family contracted during the evolution process, while the FaPP2C gene family of pineapple strawberry was the opposite. It is speculated that this phenomenon is related to the chromosome number or genome size of woodland and pineapple strawberries.

The PP2C gene family members in most plants such as *A. thaliana* [29], rice [29], wheat [50] and cucumber [51] were divided into 13 subfamilies by systematic cluster analysis. In this study, the *PP2C* genes of *A. thaliana*, woodland, and pineapple strawberries were divided into 11 subfamilies (Figure 1). This result was different from the above species. FaPP2C was present in ten subfamilies except for subfamily B, while FvPP2C was present in nine subfamilies, except for A and B. In addition, PP2C proteins of the same species tend to form independent branches in the same subfamily. The diversity of the exon/intron structure, position, and conserved motif had important effects on the gene family function [51]. Accordingly, we studied the gene structure and protein conserved motifs of *FvPP2C* and *FaPP2C* according to their phylogenetic relations (Supplementary Figures S1 and S2). The results showed that there were significant differences in gene structure between the woodland and pineapple strawberries, with *FvPP2C* containing 1–15 exons and *FaPP2C* containing 1–35 exons. Nonetheless, there were some similarities in the length of exons of the same species within the same subfamily. Previous studies on *Brachypodium distachyon* have shown that many intron-deletion genes exist in the *PP2C* gene [13]. Similar results were found in the study of woodland and pineapple strawberries in this paper. We also found that the composition and distribution of conserved motifs within the same subfamily were similar. Moreover, the amino acid sequence of motif 4 was consistent between the two species (Supplementary Figure S3), suggesting that motif 4 played a stable and important role in the evolution of strawberry. This conserved motif was also found in cucumber studies [52], and it was presumed that the motif contained the conserved domain of PP2C.

Chromosomal localization analysis and collinear analysis are very helpful to understand the mechanism of gene amplification. In this study, both *FvPP2C* and *FaPP2C* were located on chromosomes, among which *FvPP2C* was distributed on seven chromosomes and *FaPP2C* was distributed on 28 chromosomes (Figure 2). Combined with the collinearity analysis between the two species, it is speculated that the whole genome duplication is the main reason for the abundance of *PP2C* genes in pineapple strawberry (Figure 3C). In addition, the *PP2C* genes showed aggregation on the chromosomes of woodland and pineapple strawberries, and the result was similar to that of paper mulberry [53]. Gene replication is the main driving force for the amplification of the gene family, which could obtain new functions and evolution [54]. Gene replication involves fragment replication, tandem replication, and genome replication, as above-mentioned, and fragment replication is more conducive for maintaining gene function than tandem replication [55]. It was found that there was fragment replication of the PP2C gene in both woodland and pineapple strawberries (Figure 3A,B), and similar results were found in *A. thaliana* [29], cucumber [52], and paper mulberry [53].

Gene selection pressure and codon bias also contribute to understanding the evolutionary relationship. In our work, we estimated the Ka, Ks, and Ka/Ks ratios of the collinearity of *FaPP2C* and *FvPP2C*, respectively (Figure 4). The majority of the *FvPP2C* paralogous pairs showed less than 1.00 Ka/Ks ratios, but the number of homologous gene pairs with Ka/Ks ratios greater than 1.00 and less than 1.00 tended to be consistent in pineapple strawberry. These results indicate that *PP2C* of woodland strawberry mainly underwent purification selection, but there were both purification selection and positive selection effects in the evolution process of the *PP2C* of pineapple strawberry. The conservation of woodland strawberry was higher than pineapple strawberry. The result in woodland strawberry was similar to those in cucumbers and *Brassica rapa* [56]. Furthermore, the codon bias analysis of *FvPP2C* and *FaPP2C* (Figure 5 and Figure 6) showed that the codon bias of strawberry *PP2C* genes was weak, but the *PP2Cs* in subfamily K showed a relatively strong codon bias.

Subcellular localization prediction can provide basic information for the study of protein biological function. Both FvPP2C and FaPP2C were expressed mostly in the nucleus, cytoplasm, and chloroplast (Supplementary Tables S1 and S2). Similar results were found in the cucumber study [52]. Therefore, we speculated that the PP2C protein was involved in respiration, photosynthesis, growth, and development of strawberry. When plants suffer from abiotic and adverse stresses outside such as drought, high temperature, low temperature, and salinity, stress signals, through a series of signal transduction, integrate into the signal factor and activate the corresponding transcription factors. Coupled with the corresponding gene of *cis*-acting, the corresponding gene expression and response to adverse stress are initiated [57,58]. For example, ABA response elements (ABREs) can respond to ABA, drought, and salt stress [59]. Drought response element (DRE) responds to plant drought stress [60]. LTR is involved in low-temperature response and regulation [61]. MYB can respond to various stress signals and stress regulation and regulate the response of corresponding genes [62]. We analyzed the upstream 2kb *cis*-acting elements of 56 *FvPP2C* (Figure 7) and 228 *FaPP2C* (Figure 8) genes, and found that each *PP2C* contained ABRE, ERE, DRE, and other acting elements, indicating that the PP2C gene family was significantly related to the regulation of plant stress signals. All of the acting elements were divided into four categories: light response elements, hormone response elements, defense and stress response elements, and growth and development response elements. Among them, light responsive elements accounted for a large proportion of *FvPP2C* and *FaPP2C*, which further demonstrated the photoregulated reaction of the PP2C protein in strawberry. In addition, this study found that genes located in subgroup K played important roles in the ABA and MeJA responses. Cao et al. also found many action elements related to plant stress [12]. The same phenomenon has been found in *A. thaliana*, rice, foxtail millet, and *B. distachyum* [18].

Previous studies have confirmed that the expression levels of the *PP2C* subfamily A in *A. thaliana* and rice under ABA stress have significantly changed [63]. In subfamily A of *AtPP2C*, seven genes were considered as negative regulators of the ABA-mediated signaling pathway [14,29,50]. Four *PtPP2Cs* in subgroup A responded to drought, cold, and high-salt stress, and the expression of *PtPP2Cs* was the highest under drought [64]. *AtPP2C-G1* is a positive regulator in the ABA pathway and response to hypersalt stress [65]. *A. thaliana* overexpressing *AtPP2C2* showed a significantly improved response to ABA stress [66]. In grape, *VvPP2C02* can strongly respond to ABA, high salt, and drought stress [19]. Here, qRT-PCR was used to analyze the expression of 56 *FvPP2Cs* in woodland strawberry under ABA, NaCl, and PEG stress treatment (Figure 9). The results showed that only 30.36% of *FvPP2Cs* significantly increased under ABA stress treatment, so it is speculated that these genes are involved in ABA signal response as positive regulatory factors. The expression of most *FvPP2Cs* was inhibited after ABA stress, and these genes were presumed to be negative regulators in ABA signaling and abiotic stress. In addition, researchers have confirmed that *PP2Cs* play a negative regulatory role in the signaling pathway of ABA in model plants, indicating that *FvPP2Cs* has a certain degree of conservatism in the signal transduction pathway [67]. In wheat, the expression of *TaPP2C59* was significantly inhibited by ABA and hypersaline stress, and it may be involved in ABA signaling and abiotic stress response as a negative regulator [68]. Maize *ZmPP2C* is a negative regulator of salt and drought stress responses [69]. The expression of most *FvPP2Cs* genes was significantly inhibited under high salt and drought stress, and it was speculated that these genes acted as a negative regulator. In addition, the expression of *FvPP2C18* gene in strawberry was upregulated under ABA, high salt, and drought stress treatment, suggesting a positive regulatory role in ABA signaling and abiotic stress response mechanisms. However, the specific function needs to be further verified.

## 4. Materials and Methods

### 4.1. Plant Materials and Treatment

The tissue culture seedlings of *F. vesca* were selected for RT-qPCR analysis and materials were grown in MS tissue culture medium supplemented with 30 g·L^−1^ sucrose, 6 g·L^−1^ agar, 0.1 mg·L^−1^ 6-BA, and 0.2 mg·L^−1^ IAA for 16 h/8 h (day/night) cycle at 25 °C/20 °C. After 40 days of subculture, tissue culture seedlings with strong growth, consistent growth, and no pollution were selected for the experimental treatments including 10% PEG, 100 μM ABA, and 200 mM NaCl treated for 2 h, 12 h, and 24 h. Equal volume water treatment was used as the control. Plant leaves were mixed for sampling and three biological repeats were established. Leaf samples were immediately frozen in liquid nitrogen and stored at −80 °C for RNA extraction.

### 4.2. Extraction and Quality Control of RNA from Strawberry Leaves

RNA extraction was carried out using the plant-extraction kit RNAplant-RTR2303 (Real-Times Biotechnology Co., Ltd., Beijing, China) according to the operating instructions. RNA quality and quantity were determined using a Pultton P200 Micro Volume Spectrophotometer (Pultton Technology Ltd., San Jose, CA, USA). RNA was stored at −80 °C for further analysis.

### 4.3. Identification of the PP2C Gene Family Members in Strawberry

By using the conserved amino acid sequences of *PP2C* genes obtained from *A. thaliana* and rice, Blastp homology was performed in the woodland strawberry database (https://www.rosaceae.org/organism/24344, accessed on 1 October 2022) and the pineapple strawberry database (https://www.rosaceae.org/organism/24345, accessed on 5 October 2022), respectively, to screen the PP2C gene family information of woodland and pineapple strawberries. Candidate genes were screened by DNAMAN to avoid duplication, and genes containing specific domains of PP2C (registration number: PF00481) were screened by HMMER (https://www.ebi.ac.uk/Tools/hmmer/, accessed on 10 October 2022) [70]. The physicochemical properties of the theoretical isoelectric point (pI) and molecular weight were analyzed based on ExPASy (http://web.expasy.org/, accessed on 16 October 2022) [71]. Online software WoLF PSORT (http://www.genscript.com/wolf-psort.html, accessed on 21 October 2022) was used to predict subcellular localization [72].

### 4.4. Evolution Relationship of PP2C Gene Family

MEGA 11.0 and ClustalX software were used to construct a phylogenetic tree for the PP2C protein sequences of woodland strawberry, pineapple strawberry, and *A. thaliana* [23]. Maximum parsimony was adopted, and the bootstrap value was set to be equal to 1000. Image was beautified by iTOL (https://itol.embl.de/, accessed on 28 October 2022).

### 4.5. Gene Structure and Protein Conserved Motif Analysis

The gene structure of *FvPP2C* and *FaPP2C* was analyzed by GSDS (http://gsds.cbi.pku.edu.cn/, accessed on 5 November 2022) [73]. MEME (http://meme-suite.org/tools/meme, accessed on 13 November 2022) was used for the conserved motif analysis [74]. The maximum motif number was set as 8, and the remaining parameters were the default values.

### 4.6. Chromosomal Location and Collinearity Analysis

We downloaded the genome files of woodland strawberry, pineapple strawberry, and *A. thaliana* via the Ensemble database (https://asia.ensembl.org/index.html, accessed on 20 November 2022) and Phytozome database (https://phytozome-next.jgi.doe.gov/, accessed on 1 December 2022). TBtools was used to extract the chromosome information and draw chromosomal mapping of the target genes. The analysis and mapping of collinear gene pairs were also conducted with TBtools [75].

### 4.7. Selective Pressure Analysis and Codon Usage Index Analysis

The nonsynonymous substitution rate and synonymous substitution rate of *PP2C* collinear genes in woodland strawberry and pineapple strawberry were calculated by DNAsp 5.0 and mapped by Origin 9.0. CodonW software was used to analyze the codon usage characteristics of strawberry. The main parameters were acquired by employing CDS sequences. These parameters included A3s, G3s, C3s, and T3s (synonymous codon corresponding base frequency on the third), the codon adaptation index (CAI), codon bias index (CBI), frequency of optimal codons (FOP), effective number of codon (ENc), amount of the third codon (G+C) (GC3s), count of genes (G+C) (GC), triple codon, and analyzed the correlation of these parameters.

### 4.8. Cis-Acting Elements Analysis

The upstream 2 kb sequences of the *PP2C* gene initiation codon (ATG) were extracted by TBtools. The extracted sequences were submitted to PlantCARE (http://bioinformatics.psb.ugent.be/webtools/plantcare/html/, accessed on 15 December 2022) for *cis*-acting element analysis [76].

### 4.9. qRT-PCR Analysis

The fluorescent quantitative primers of *FvPP2Cs* were designed in the online primer design tool Primer3 (http://bioinfo.ut.ee/primer3-0.4.0/, accessed on 23 December 2022) (Supplementary Table S3) and synthesized by Sangon Biotech (Shanghai, China) Co., Ltd. cDNA synthesis was performed using a Primer Reverse Transcriptase Kit (Takara Bio, Shiga, Japan), and quantitative PCR was performed using the SYBR (concentration: 2x) Primer Ex Taq^TM^II (TaKaRa) Kit specifications, and conducted by using a Light Cycler^®^ 96 Real-Time PCR System (Roche, Switzerland). The *GAPDH* of strawberry was used as the internal reference gene. The reaction system was 20 μL and comprised 1.5 μL of cDNA, 2 μL of upstream and downstream primers, 10 μL of SYBR, and 6.5 μL of ddH_2_O. The reaction procedure was as follows: 95 °C for 30 s, 40 cycles of 95 °C for 10 s, 60 °C for 30 s, and 72 °C for 30 s. The experiment was repeated three times. After the reaction to fluorescence value change curve and melting curve analysis, the 2^−ΔΔCT^ method was used to calculate the relative expression of genes [77].

### 4.10. Test Data Statistics and Analysis

Test data were processed by Excel 2010. TBtools was used to draw the relative expression map of genes.

## 5. Conclusions

In this study, 56 *FvPP2C* and 228 *FaPP2C* genes were identified from woodland strawberry and pineapple strawberry, respectively. Compared with diploid woodland strawberry, the PP2C gene family of pineapple strawberry had large differences in the gene size and relatively weak conservation. The whole genome replication was the main cause of the *PP2C* gene quantity amplification in pineapple strawberry. The analysis of *cis*-acting elements and the expression pattern of *PP2C* genes in woodland strawberry under different stress indicate that there are a series of potential mechanisms of the PP2C gene family in strawberry to abiotic stress, which provide a reference for understanding the PP2C gene family and its functions in woodland and pineapple strawberries.

## Data Availability

Data will be made available on request.

## Supplementary Materials

The following supporting information can be downloaded at: https://www.mdpi.com/article/10.3390/ijms24044049/s1.

## Abbreviations

PP2C: protein phosphatase 2C; ABA: abscisic acid; SA: salicylic acid; MeJA: methyl jasmonate; GA: gibberellin; PKs: protein kinases; PPs: protein phosphatases; Ser: serine; Thr: threonine; Tyr: tyrosine; STPs: serine/threonine phosphatases; PTPs: protein tyrosine phosphatases; DSPTPs: protein dual specificity phosphatases; PPM: phosphoprotein metal phosphatases; PPP: phosphoprotein phosphatases; PYR/PYL/RCAR: ABA receptor protein; SnRK2s: SNF1-associated protein kinase 2; MAPK: mitogen-activated protein kinase; Ks: synonymous substitution; Ka: non-synonymous substitution; ENc: the effective number of codon; CAI: codon adaptation index; CBI: codon bias index; FOP: frequency of optimal codons; GC3s: the amount of the third codon (G+C); GC: the count of genes (G+C); RSCU: the relative synonymous codon usage; qRT-PCR: quantitative real-time PCR; ABREs: ABA response elements; DRE: drought response element; pI: theoretical isoelectric point.
