# Supplementary material for: Genome-Wide Identification and Abiotic Stress Response Analysis of PP2C Gene Family in Woodland and Pineapple Strawberries
Source: Int J Mol Sci. 2023 Feb 17;24(4):4049. doi: 10.3390/ijms24044049 (PMC9961684; doi:10.3390/ijms24044049)
Supplement: Supplementary file 1 [file ijms-24-04049-s001.zip › Captions for Figures S1-S3.pdf]

Supplementary Figure S1. Gene structure analysis and conserved motif prediction of *FvPP2Cs*. Note: (A) The results of gene structure analysis of polypeptide sequences cluster analysis tree using maximum parsimony method. CDS (coding sequence) regions were marked by pink boxes, upstream and downstream regions with blue boxes, and introns with black lines. (B) The motifs represented by the boxes with different colors refer to the legend on the right. The scales at the bottom are used to measure the length of the sequence.

Supplementary Figure S2. Gene structure analysis and conserved motif prediction of *FvPP2Cs*. Note: (A) The results of gene structure analysis of polypeptide sequences cluster analysis tree using maximum parsimony method. CDS regions were marked by yellow boxes, upstream and downstream regions with green boxes, and introns with black lines. (B) The motifs represented by the boxes with different colors refer to the legend on the right. The scales at the bottom are used to measure the length of the sequence.

Supplementary Figure S3. The conserved motif logos of PP2Cs in woodland and pineapple strawberries.
