# Supplementary material for: Genome-Wide Identification and Abiotic Stress Response Analysis of PP2C Gene Family in Woodland and Pineapple Strawberries
Source: Int J Mol Sci. 2023 Feb 17;24(4):4049. doi: 10.3390/ijms24044049 (PMC9961684; doi:10.3390/ijms24044049)
Supplement: Supplementary file 1 [file ijms-24-04049-s001.zip › Supplementary Table S1.pdf]

Supplementary Table S1 The physicochemical properties of *FvPP2Cs* in woodland strawberry

Group	Gene name	Sequence ID	Length (aa)	MW (Da)	pI	Full length (bp)	GRAVY	Subcellular localization
C	*FvPP2C29*	FvH4_4g17950.1	499	54451.08	4.89	3452	-0.244	Nuclear
	FvPP2C38	FvH4_5g21630.1	787	86512.58	5.37	5658	-0.531	Chloroplast
	FvPP2C45	FvH4_6g13730.1	667	74684.97	5.67	2975	-0.521	Nuclear
	FvPP2C21	FvH4_3g35990.1	720	80511.07	5.58	3381	-0.519	Chloroplast
D	*FvPP2C08*	FvH4_2g04560.1	383	42632.62	8.71	2756	-0.285	Chloroplast
	FvPP2C06	FvH4_1g17120.1	373	42010.91	5.97	3173	-0.258	Cytoplasm
	FvPP2C31	FvH4_4g19920.1	383	42097.94	6.52	3752	-0.194	Chloroplast
	FvPP2C47	FvH4_6g22310.1	383	43217.32	6.47	3240	-0.278	Cytoplasm
	FvPP2C46	FvH4_6g14920.1	380	42635.78	9.3	2677	-0.327	Chloroplast
	FvPP2C51	FvH4_7g20640.1	385	42903.49	9.2	4119	-0.23	Chloroplast
	FvPP2C09	FvH4_2g15570.1	395	43837.76	8.45	4929	-0.276	Nuclear
E	*FvPP2C05*	FvH4_1g11150.1	393	43461.53	8.26	3944	-0.221	Chloroplast
	FvPP2C33	FvH4_4g25730.1	542	58832.58	8.71	3280	-0.531	Nuclear
	FvPP2C53	FvH4_7g24650.1	373	40224.8	6.67	1850	-0.242	Nuclear
	FvPP2C23	FvH4_3g38990.1	380	41170.62	8.2	2302	-0.364	Nuclear
F	*FvPP2C12*	FvH4_2g29850.1	377	41353.61	5.35	2765	-0.349	Chloroplast
	FvPP2C32	FvH4_4g20910.1	385	42495.07	5.34	2376	-0.193	Nuclear
	FvPP2C07	FvH4_1g26700.1	387	42873.46	4.85	3096	-0.214	Nuclear
	FvPP2C39	FvH4_5g25660.1	385	42210.07	5.57	3237	-0.236	Cytoplasm
	FvPP2C04	FvH4_1g10220.1	390	42156.71	5.61	2946	-0.252	Nuclear

G	*FvPP2C10*	FvH4_2g16790.1	385	41957.39	5.31	3286	-0.241	Nuclear
	FvPP2C11	FvH4_2g18940.1	658	72746.13	5.8	1977	-0.124	Cytoplasm
	FvPP2C48	FvH4_6g28470.1	475	51354.32	5.39	7385	-0.633	Nuclear
	FvPP2C16	FvH4_2g41190.1	365	39912.75	4.95	6175	-0.414	Chloroplast
	FvPP2C37	FvH4_5g16840.1	329	35988.04	8.19	3262	-0.312	Cytoplasm
	FvPP2C56	FvH4_7g32030.1	420	45564.19	5.44	3149	-0.2	Chloroplast
	FvPP2C54	FvH4_7g31190.1	345	37723.4	5.75	4141	-0.349	Chloroplast
H	*FvPP2C17*	FvH4_2g41410.1	292	31463.87	5.04	4309	-0.342	Nuclear
	FvPP2C25	FvH4_4g03890.1	271	30749.07	9.24	2296	-0.572	Nuclear
	FvPP2C18	FvH4_3g10800.1	273	30328.15	4.9	1090	-0.338	Cytoplasm
	FvPP2C40	FvH4_5g37270.1	282	30902.67	7.09	5076	-0.158	Chloroplast
	FvPP2C19	FvH4_3g19640.1	282	31332.55	5.9	2807	-0.408	Nuclear
I	*FvPP2C28*	FvH4_4g14950.1	597	66286.4	6.36	6104	-0.324	Chloroplast
	FvPP2C15	FvH4_2g34570.1	432	46820.93	5.01	4165	-0.324	Chloroplast
	FvPP2C43	FvH4_6g07050.1	426	45973.7	5.99	3483	-0.121	Chloroplast
	FvPP2C35	FvH4_4g26880.1	429	46053.43	8.27	3450	-0.238	Chloroplast
J	*FvPP2C27*	FvH4_4g13960.1	432	45879.01	5.14	4036	-0.379	Nuclear
	FvPP2C01	FvH4_1g05740.1	1080	119808.08	4.88	6991	-0.232	Nuclear
	FvPP2C42	FvH4_6g06970.1	376	41950.44	5.76	3804	-0.369	Nuclear
	FvPP2C30	FvH4_4g18870.1	377	41911.93	8.95	2826	-0.302	Nuclear
	FvPP2C14	FvH4_2g32170.1	336	36465.2	5.09	1674	-0.139	Cytoplasm
	FvPP2C44	FvH4_6g10560.1	453	50272.18	4.99	2701	-0.532	Chloroplast

	FvPP2C50	FvH4_6g45040.1	470	52006.45	5.45	4445	-0.488	Chloroplast
	FvPP2C24	FvH4_3g41300.1	534	58715.13	5.05	4085	-0.391	Nuclear
	FvPP2C41	FvH4_6g00430.1	497	54676.21	5.21	4105	-0.451	Chloroplast
	FvPP2C26	FvH4_4g12270.1	424	45688.02	7.56	5138	-0.123	Cytoplasm
	FvPP2C03	FvH4_1g08010.1	476	52094.86	5.55	2978	-0.336	Chloroplast
	FvPP2C13	FvH4_2g31450.1	446	47853.84	4.34	4378	-0.034	Nuclear
	FvPP2C49	FvH4_6g34170.1	552	59486.52	4.94	3684	-0.088	Extracellular matrix
	FvPP2C34	FvH4_4g25760.1	551	59394.42	4.96	5791	-0.063	Chloroplast
	FvPP2C02	FvH4_1g06040.1	371	40546.6	5.04	4333	-0.276	Chloroplast
K	*FvPP2C55*	FvH4_7g31810.1	389	42639.17	5.66	2014	-0.312	Nuclear
	FvPP2C36	FvH4_5g04500.1	350	37509.95	5.24	1053	-0.276	Chloroplast
	FvPP2C52	FvH4_7g23060.1	422	45402.3	5.99	2344	-0.356	Chloroplast
	FvPP2C22	FvH4_3g37010.1	397	43679.28	5.33	2213	-0.393	Chloroplast
	FvPP2C20	FvH4_4g18870.1	377	41911.93	8.95	2826	-0.302	Chloroplast
