# Supplementary material for: Genome-Wide Identification and Abiotic Stress Response Analysis of PP2C Gene Family in Woodland and Pineapple Strawberries
Source: Int J Mol Sci. 2023 Feb 17;24(4):4049. doi: 10.3390/ijms24044049 (PMC9961684; doi:10.3390/ijms24044049)
Supplement: Supplementary file 1 [file ijms-24-04049-s001.zip › Supplementary Table S2.pdf]

Supplementary Table S2 The physicochemical properties of *FaPP2Cs* in pineapple strawberry

Group	Gene name	Sequence ID	Length (aa)	MW (Da)	pI	Full length (bp)	GRAVY	Subcellular localization
A	*FaPP2C213*	snap_masked-Fvb1-4-processed-gene-122.24-mRNA-1	314	34142.78	6.75	1385	-0.041	Nuclear
C	*FaPP2C124*	maker-Fvb4-2-augustus-gene-95.40-mRNA-1	504	55061.83	4.94	3572	-0.251	Nuclear
	FaPP2C136	maker-Fvb4-4-augustus-gene-107.30-mRNA-1	504	55005.77	4.86	3649	-0.245	Nuclear
	FaPP2C129	maker-Fvb4-3-augustus-gene-192.33-mRNA-1	504	54954.65	4.86	3434	-0.240	Nuclear
	FaPP2C127	maker-Fvb4-3-augustus-gene-138.38-mRNA-1	504	54968.67	4.86	3372	-0.241	Nuclear
	FaPP2C145	maker-Fvb5-2-augustus-gene-127.34-mRNA-1	787	86375.41	5.34	5992	-0.528	Nuclear
	FaPP2C150	maker-Fvb5-4-augustus-gene-109.32-mRNA-1	787	86370.49	5.41	5667	-0.519	Chloroplast
	FaPP2C148	maker-Fvb5-3-augustus-gene-152.35-mRNA-1	787	86599.66	5.38	5736	-0.540	Chloroplast
	FaPP2C142	maker-Fvb5-1-augustus-gene-138.41-mRNA-1	782	86015.97	5.33	5645	-0.537	Chloroplast
	FaPP2C168	maker-Fvb6-2-snap-gene-192.61-mRNA-1	685	76972.69	5.86	2927	-0.498	Nuclear
	FaPP2C178	maker-Fvb6-3-snap-gene-306.46-mRNA-1	665	74580.88	5.65	3208	-0.519	Nuclear
	FaPP2C155	maker-Fvb6-1-augustus-gene-270.38-mRNA-1	667	74598.89	5.78	3788	-0.500	Nuclear
	FaPP2C186	maker-Fvb6-4-augustus-gene-97.36-mRNA-1	668	74777.14	5.88	3461	-0.523	Nuclear
	FaPP2C111	maker-Fvb3-4-augustus-gene-64.43-mRNA-1	718	80356.91	5.58	4475	-0.521	Chloroplast
	FaPP2C100	maker-Fvb3-3-augustus-gene-234.19-mRNA-1	718	80310.90	5.58	3793	-0.515	Chloroplast
	FaPP2C93	maker-Fvb3-1-augustus-gene-62.13-mRNA-1	720	80432.96	5.49	3916	-0.502	Chloroplast
	FaPP2C97	maker-Fvb3-2-augustus-gene-253.31-mRNA-1	714	80058.61	5.58	3313	-0.515	Chloroplast
D	*FaPP2C162*	maker-Fvb6-1-snap-gene-208.65-mRNA-1	186	21196.24	7.12	2599	-0.418	Nuclear
	FaPP2C154	maker-Fvb6-1-augustus-gene-201.46-mRNA-1	383	43200.38	6.87	3334	-0.271	Cytoplasm
	FaPP2C18	maker-Fvb6-4-augustus-	383	43203.30	6.47	2853	-0.283	Cytoplasm

1	gene-162.31-mRNA-1						
FaPP2C163	maker-Fvb6-2-augustus-gene-124.24-mRNA-1	383	43276.33	6.47	3178	-0.301	Cytoplasm
FaPP2C170	maker-Fvb6-3-augustus-gene-215.29-mRNA-1	397	44805.28	6.40	3274	-0.234	Cytoplasm
FaPP2C217	snap_masked-Fvb2-3-processed-gene-210.15-mRNA-1	417	46418.00	8.90	6192	-0.251	Chloroplast
FaPP2C218	snap_masked-Fvb2-4-processed-gene-23.22-mRNA-1	422	46845.35	8.17	2987	-0.254	Chloroplast
FaPP2C214	snap_masked-Fvb2-1-processed-gene-18.24-mRNA-1	423	46873.38	7.74	2607	-0.248	Chloroplast
FaPP2C65	maker-Fvb1-4-augustus-gene-88.43-mRNA-1	449	50741.11	5.93	4630	-0.129	Cytoplasm
FaPP2C39	maker-Fvb1-1-augustus-gene-181.33-mRNA-1	448	50654.01	5.77	3458	-0.138	Cytoplasm
FaPP2C46	maker-Fvb1-2-augustus-gene-109.34-mRNA-1	449	50860.17	5.74	3333	-0.149	Cytoplasm
FaPP2C59	maker-Fvb1-3-augustus-gene-97.51-mRNA-1	449	50695.96	5.83	3501	-0.144	Cytoplasm
FaPP2C126	maker-Fvb4-3-augustus-gene-125.56-mRNA-1	384	42160.01	6.50	3934	-0.190	Vacuolar
FaPP2C19	augustus_masked-Fvb4-4-processed-gene-93.12-mRNA-1	1183	132307.41	6.36	10019	-0.263	Plasma membrane
FaPP2C164	maker-Fvb6-2-augustus-gene-184.37-mRNA-1	380	42631.80	9.30	2916	-0.319	Mitochondrial
FaPP2C226	snap_masked-Fvb6-1-processed-gene-262.36-mRNA-1	395	44593.02	9.42	2670	-0.382	Mitochondrial
FaPP2C180	maker-Fvb6-4-augustus-gene-104.54-mRNA-1	380	42679.83	9.20	2701	-0.323	Chloroplast
FaPP2C171	maker-Fvb6-3-augustus-gene-325.48-mRNA-1	380	42651.78	9.30	2714	-0.335	Mitochondrial
FaPP2C188	maker-Fvb7-1-augustus-gene-245.60-mRNA-1	385	42893.44	9.11	7002	-0.224	Chloroplast
FaPP2C204	maker-Fvb7-3-augustus-gene-79.53-mRNA-1	385	42951.53	9.20	4168	-0.232	Chloroplast
FaPP2C200	maker-Fvb7-2-snap-gene-219.91-mRNA-1	345	38499.49	9.11	7021	-0.247	Chloroplast
FaPP2C198	maker-Fvb7-2-augustus-gene-301.38-mRNA-1	385	42903.49	9.20	4078	-0.230	Chloroplast

	FaPP2C209	maker-Fvb7-4-augustus-gene-76.42-mRNA-1	385	42937.50	9.20	2808	-0.229	Chloroplast
	FaPP2C208	maker-Fvb7-4-augustus-gene-74.49-mRNA-1	385	42937.50	9.20	2808	-0.229	Chloroplast
	FaPP2C81	maker-Fvb2-4-augustus-gene-120.19-mRNA-1	395	43853.76	8.45	4804	-0.283	Nuclear
	FaPP2C77	maker-Fvb2-3-augustus-gene-118.28-mRNA-1	395	43837.72	8.17	4987	-0.275	Nuclear
	FaPP2C72	maker-Fvb2-2-augustus-gene-108.55-mRNA-1	395	43853.81	8.45	4844	-0.263	Nuclear
	FaPP2C216	snap_masked-Fvb2-1-processed-gene-93.14-mRNA-1	416	46152.64	8.58	4659	-0.196	Chloroplast
	FaPP2C67	maker-Fvb1-4-snap-gene-52.90-mRNA-1	393	43534.58	8.55	3891	-0.243	Chloroplast
	FaPP2C45	maker-Fvb1-1-snap-gene-222.55-mRNA-1	393	43498.54	8.55	3699	-0.238	Chloroplast
	FaPP2C212	snap_masked-Fvb1-2-processed-gene-74.36-mRNA-1	428	47470.24	9.02	3801	-0.228	Chloroplast
	FaPP2C58	maker-Fvb1-3-augustus-gene-59.44-mRNA-1	393	43504.55	8.26	4209	-0.235	Chloroplast
	FaPP2C122	maker-Fvb4-2-augustus-gene-59.42-mRNA-1	542	59145.98	8.63	3506	-0.519	Nuclear
	FaPP2C139	maker-Fvb4-4-augustus-gene-58.36-mRNA-1	542	59298.23	8.82	3482	-0.537	Nuclear
	FaPP2C116	maker-Fvb4-1-augustus-gene-142.35-mRNA-1	540	59026.13	8.94	3574	-0.532	Nuclear
	FaPP2C133	maker-Fvb4-3-augustus-gene-91.57-mRNA-1	542	58888.65	8.71	3276	-0.524	Nuclear
	FaPP2C99	maker-Fvb3-2-augustus-gene-272.29-mRNA-1	380	41172.57	7.02	2029	-0.347	Chloroplast
E	*FaPP2C110*	maker-Fvb3-4-augustus-gene-46.35-mRNA-1	380	41216.64	7.93	2051	-0.362	Chloroplast
	FaPP2C102	maker-Fvb3-3-augustus-gene-255.28-mRNA-1	380	41223.53	7.51	2055	-0.377	Chloroplast
	FaPP2C91	maker-Fvb3-1-augustus-gene-43.30-mRNA-1	380	40904.14	7.49	2539	-0.361	Chloroplast
	FaPP2C189	maker-Fvb7-1-augustus-gene-260.26-mRNA-1	412	44853.14	8.68	2233	-0.391	Nuclear
	FaPP2C33	augustus_masked-Fvb7-2-processed-gene-243.9-mRNA-1	1038	114863.19	6.99	8943	-0.128	Chloroplast
	FaPP2C38	augustus_masked-Fvb7-4-	2016	226678.0	5.43	13624	-0.186	Chloroplast

		processed-gene-45.11-mRNA-1		9				
	FaPP2C36	augustus_masked-Fvb7-3-processed-gene-59.9-mRNA-1	2106	236904.86	5.43	13793	-0.169	Chloroplast
	FaPP2C09	augustus_masked-Fvb2-4-processed-gene-146.8-mRNA-1	658	72942.40	5.84	1977	-0.141	Cytoplasm
	FaPP2C07	augustus_masked-Fvb2-2-processed-gene-85.6-mRNA-1	658	72776.15	5.80	1977	-0.128	Cytoplasm
	FaPP2C82	maker-Fvb2-4-augustus-gene-129.28-mRNA-1	385	41942.31	5.31	3628	-0.251	Nuclear
	FaPP2C68	maker-Fvb2-1-augustus-gene-102.38-mRNA-1	385	41991.45	5.24	3179	-0.226	Nuclear
	FaPP2C57	maker-Fvb1-3-augustus-gene-54.51-mRNA-1	390	42166.81	5.61	3793	-0.242	Nuclear
	FaPP2C51	maker-Fvb1-2-augustus-gene-70.48-mRNA-1	390	42245.72	5.35	3015	-0.260	Nuclear
	FaPP2C40	maker-Fvb1-1-augustus-gene-227.48-mRNA-1	390	42207.84	5.70	2980	-0.258	Nuclear
	FaPP2C64	maker-Fvb1-4-augustus-gene-47.35-mRNA-1	390	42133.68	5.43	2943	-0.250	Nuclear
F	*FaPP2C22*	augustus_masked-Fvb5-2-processed-gene-164.7-mRNA-1	248	27111.79	5.56	2587	-0.184	Cytoplasm
	FaPP2C151	maker-Fvb5-4-augustus-gene-144.29-mRNA-1	436	48330.23	5.78	3766	-0.236	Nuclear
	FaPP2C147	maker-Fvb5-3-augustus-gene-118.34-mRNA-1	391	43087.21	5.80	3003	-0.232	Nuclear
	FaPP2C143	maker-Fvb5-1-augustus-gene-175.42-mRNA-1	388	42816.27	5.98	2869	-0.145	Cytoplasm
	FaPP2C211	snap_masked-Fvb1-1-processed-gene-266.33-mRNA-1	370	40895.80	4.81	2374	-0.355	Cytoskeleton
	FaPP2C75	maker-Fvb2-2-augustus-gene-23.35-mRNA-1	377	41353.61	5.35	3615	-0.349	Chloroplast
	FaPP2C04	augustus_masked-Fvb2-1-processed-gene-197.12-mRNA-1	378	41498.79	5.27	3859	-0.336	Chloroplast
	FaPP2C80	maker-Fvb2-3-augustus-gene-9.48-mRNA-1	377	41479.95	5.65	5200	-0.330	Chloroplast
	FaPP2C84	maker-Fvb2-4-augustus-gene-211.36-mRNA-1	378	41504.87	5.44	3415	-0.332	Chloroplast

	FaPP2C224	snap_masked-Fvb4-4-processed-gene-88.24-mRNA-1	413	45742.55	5.21	2316	-0.236	Nuclear
	FaPP2C123	maker-Fvb4-2-augustus-gene-89.36-mRNA-1	385	42393.94	5.20	2697	-0.174	Nuclear
	FaPP2C62	maker-Fvb1-4-augustus-gene-171.15-mRNA-1	387	42831.42	4.85	3740	-0.213	Cytoplasm, Nuclear
	FaPP2C47	maker-Fvb1-2-augustus-gene-207.16-mRNA-1	387	42812.46	4.72	6357	-0.153	Cytoplasm
	FaPP2C44	maker-Fvb1-1-augustus-gene-84.21-mRNA-1	296	32985.38	4.85	3018	-0.158	Nuclear
	FaPP2C54	maker-Fvb1-3-augustus-gene-201.15-mRNA-1	381	42340.94	4.78	4045	-0.177	Nuclear
	FaPP2C152	maker-Fvb5-4-augustus-gene-74.38-mRNA-1	260	28415.76	6.31	4112	0.127	Cytoplasm
	FaPP2C146	maker-Fvb5-2-snap-gene-94.19-mRNA-1	423	46961.12	8.89	3325	-0.183	Cytoplasm
	FaPP2C141	maker-Fvb5-1-augustus-gene-103.47-mRNA-1	329	35957.95	8.19	3894	-0.320	Cytoplasm
	FaPP2C210	maker-Fvb7-4-augustus-gene-9.78-mRNA-1	146	16180.22	4.62	3068	-0.070	Chloroplast
	FaPP2C201	maker-Fvb7-2-snap-gene-288.90-mRNA-1	429	46516.32	5.33	3084	-0.158	Chloroplast
	FaPP2C194	maker-Fvb7-1-augustus-gene-313.69-mRNA-1	163	18090.34	4.77	3029	-0.201	Chloroplast
	FaPP2C192	maker-Fvb7-1-augustus-gene-297.45-mRNA-1	163	18090.34	4.77	3091	-0.201	Chloroplast
G	*FaPP2C06*	augustus_masked-Fvb2-2-processed-gene-62.1-mRNA-1	1077	121856.44	5.97	10534	-0.355	Chloroplast
	FaPP2C08	augustus_masked-Fvb2-3-processed-gene-101.4-mRNA-1	1090	123460.09	5.89	10867	-0.376	Cytoplasm
	FaPP2C83	maker-Fvb2-4-augustus-gene-171.27-mRNA-1	1068	120968.22	5.89	10546	-0.381	Chloroplast
	FaPP2C03	augustus_masked-Fvb2-1-processed-gene-150.0-mRNA-1	1081	122667.33	5.85	10912	-0.356	Cytoplasm
	FaPP2C177	maker-Fvb6-3-snap-gene-162.52-mRNA-1	408	44810.62	5.07	7326	-0.389	Nuclear
	FaPP2C161	maker-Fvb6-1-snap-gene-166.61-mRNA-1	488	52853.24	5.39	7777	-0.574	Nuclear
	FaPP2C227	snap_masked-Fvb6-3-processed-gene-124.24-	101	10979.42	4.40	306	-0.044	Chloroplast

		mRNA-1						
	FaPP2C225	snap_masked-Fvb6-1-processed-gene-130.27-mRNA-1	128	14128.99	4.59	1327	-0.227	Mitochondrial
	FaPP2C87	maker-Fvb2-4-snap-gene-265.150-mRNA-1	540	60185.26	7.22	6075	-0.523	Nuclear
	FaPP2C71	maker-Fvb2-1-snap-gene-263.74-mRNA-1	530	58877.69	6.56	6110	-0.534	Nuclear
	FaPP2C219	snap_masked-Fvb2-4-processed-gene-265.51-mRNA-1	313	33831.60	4.93	4374	-0.312	Nuclear
	FaPP2C215	snap_masked-Fvb2-1-processed-gene-264.38-mRNA-1	305	33035.78	5.00	4314	-0.298	Nuclear
	FaPP2C37	augustus_masked-Fvb7-4-processed-gene-14.2-mRNA-1	350	38180.76	5.51	4008	-0.353	Chloroplast
	FaPP2C35	augustus_masked-Fvb7-3-processed-gene-25.7-mRNA-1	358	38954.64	5.51	4158	-0.319	Chloroplast
	FaPP2C199	maker-Fvb7-2-augustus-gene-320.49-mRNA-1	360	39449.29	5.42	4088	-0.345	Chloroplast
	FaPP2C196	maker-Fvb7-2-augustus-gene-283.54-mRNA-1	360	39449.29	5.42	4178	-0.345	Chloroplast
	FaPP2C32	augustus_masked-Fvb7-1-processed-gene-310.11-mRNA-1	309	33876.60	4.91	8458	-0.428	Cytoplasm
H	*FaPP2C31*	augustus_masked-Fvb7-1-processed-gene-294.13-mRNA-1	309	33786.47	4.90	4513	-0.414	Cytoplasm
	FaPP2C220	snap_masked-Fvb4-1-processed-gene-26.17-mRNA-1	279	31614.20	9.27	2225	-0.563	Nuclear
	FaPP2C121	maker-Fvb4-2-augustus-gene-228.29-mRNA-1	279	31514.98	9.02	2192	-0.565	Nuclear
	FaPP2C90	maker-Fvb3-1-augustus-gene-263.56-mRNA-1	1120	124246.12	5.35	6939	-0.246	Plasma membrane
	FaPP2C89	maker-Fvb3-1-augustus-gene-263.55-mRNA-1	1731	193107.62	5.80	9035	-0.449	Plasma membrane
	FaPP2C109	maker-Fvb3-4-augustus-gene-235.49-mRNA-1	1726	192685.63	5.73	9304	-0.482	Plasma membrane
	FaPP2C106	maker-Fvb3-3-snap-gene-40.51-mRNA-1	194	21490.07	5.04	969	-0.443	Cytoplasm
	FaPP2C14	maker-Fvb5-3-snap-gene-	303	33410.77	7.76	4625	-0.420	Chloroplast

	9	24.40-mRNA-1						
	FaPP2C24	augustus_masked-Fvb5-2-processed-gene-232.1-mRNA-1	285	31393.23	8.31	4958	-0.571	Nuclear
	FaPP2C26	augustus_masked-Fvb5-4-processed-gene-231.0-mRNA-1	282	30905.67	7.06	5151	-0.513	Chloroplast
	FaPP2C144	maker-Fvb5-1-augustus-gene-281.44-mRNA-1	282	30902.67	7.09	5112	-0.518	Nuclear
	FaPP2C95	maker-Fvb3-2-augustus-gene-109.29-mRNA-1	282	31337.52	6.07	4100	-0.426	Cytoplasm
	FaPP2C104	maker-Fvb3-3-augustus-gene-98.22-mRNA-1	282	31227.45	6.14	2807	-0.390	Cytoplasm
	FaPP2C108	maker-Fvb3-4-augustus-gene-192.19-mRNA-1	282	31332.55	5.90	2793	-0.408	Cytoplasm
	FaPP2C11	augustus_masked-Fvb3-1-processed-gene-205.3-mRNA-1	282	31316.49	5.90	2781	-0.416	Cytoplasm
	FaPP2C128	maker-Fvb4-3-augustus-gene-169.27-mRNA-1	595	65995.05	6.36	6255	-0.320	Chloroplast
	FaPP2C114	maker-Fvb4-1-augustus-gene-113.38-mRNA-1	599	66359.20	5.76	6469	-0.351	Nuclear
	FaPP2C119	maker-Fvb4-2-augustus-gene-115.46-mRNA-1	574	63290.73	5.54	6169	-0.265	Chloroplast
	FaPP2C138	maker-Fvb4-4-augustus-gene-126.41-mRNA-1	572	63194.76	5.40	6506	-0.249	Chloroplast
	FaPP2C179	maker-Fvb6-3-snap-gene-81.57-mRNA-1	189	19855.38	4.58	2021	0.162	Vacuolar
	FaPP2C221	snap_masked-Fvb4-2-processed-gene-174.21-mRNA-1	87	9255.33	4.70	2237	-0.074	Chloroplast
I								
	FaPP2C113	maker-Fvb3-4-snap-gene-87.52-mRNA-1	229	24134.84	5.17	2178	0.467	Vacuolar
	FaPP2C78	maker-Fvb2-3-augustus-gene-57.62-mRNA-1	432	46776.00	5.05	4633	-0.297	Chloroplast
	FaPP2C86	maker-Fvb2-4-augustus-gene-235.52-mRNA-1	432	46758.95	5.11	4228	-0.311	Chloroplast
	FaPP2C05	augustus_masked-Fvb2-1-processed-gene-226.19-mRNA-1	432	46787.91	5.01	4229	-0.312	Chloroplast
	FaPP2C17	augustus_masked-Fvb4-3-processed-gene-83.11-mRNA-1	447	48416.95	7.07	3673	-0.293	Chloroplast
	FaPP2C22	snap_masked-Fvb4-4-	483	52499.00	8.38	3383	-0.227	Chloroplast

	3	processed-gene-51.45-mRNA-1						
	FaPP2C117	maker-Fvb4-1-augustus-gene-148.35-mRNA-1	1536	167720.41	8.46	15524	-0.075	Plasma membrane
	FaPP2C173	maker-Fvb6-3-augustus-gene-376.32-mRNA-1	389	41773.90	5.97	3779	-0.092	Chloroplast
	FaPP2C30	augustus_masked-Fvb6-4-processed-gene-52.9-mRNA-1	397	42696.96	6.07	4335	-0.104	Chloroplast
	FaPP2C157	maker-Fvb6-1-augustus-gene-320.43-mRNA-1	396	42881.04	5.83	2856	-0.136	Nuclear
	FaPP2C28	augustus_masked-Fvb6-2-processed-gene-234.0-mRNA-1	414	44548.11	6.07	3743	-0.087	Chloroplast
	FaPP2C222	snap_masked-Fvb4-3-processed-gene-179.17-mRNA-1	422	45849.98	5.27	4141	-0.393	Nuclear
	FaPP2C115	maker-Fvb4-1-augustus-gene-122.28-mRNA-1	454	49649.47	5.77	4091	-0.361	Nuclear
	FaPP2C118	maker-Fvb4-2-augustus-gene-108.13-mRNA-1	421	45712.84	5.21	4267	-0.386	Nuclear
	FaPP2C61	maker-Fvb1-4-augustus-gene-148.26-mRNA-1	186	19850.38	4.54	2126	-0.052	Extracellular matrix
	FaPP2C137	maker-Fvb4-4-augustus-gene-118.24-mRNA-1	422	45950.14	5.36	4037	-0.404	Nuclear
	FaPP2C60	maker-Fvb1-4-augustus-gene-1.48-mRNA-1	1099	122000.83	4.88	6970	-0.205	Nuclear
	FaPP2C43	maker-Fvb1-1-augustus-gene-249.36-mRNA-1	1088	121134.45	4.80	6909	-0.248	Chloroplast
J	*FaPP2C55*	maker-Fvb1-3-augustus-gene-27.37-mRNA-1	1099	122086.94	4.92	6806	-0.211	Chloroplast
	FaPP2C53	maker-Fvb1-2-snap-gene-16.71-mRNA-1	1080	119945.14	4.87	6781	-0.246	Chloroplast
	FaPP2C49	maker-Fvb1-2-augustus-gene-28.54-mRNA-1	1081	120098.45	4.94	6705	-0.240	Chloroplast
	FaPP2C73	maker-Fvb2-2-augustus-gene-11.31-mRNA-1	336	36437.18	5.09	1787	-0.137	Cytoplasm
	FaPP2C70	maker-Fvb2-1-augustus-gene-212.54-mRNA-1	336	36534.17	4.89	2108	-0.150	Nuclear
	FaPP2C79	maker-Fvb2-3-augustus-gene-68.63-mRNA-1	336	36623.22	4.91	2367	-0.192	Nuclear
	FaPP2C10	augustus_masked-Fvb2-4-processed-gene-225.9-mRNA-1	335	36336.99	5.01	2478	-0.167	Nuclear

FaPP2C140	maker-Fvb4-4-snap-gene-100.57-mRNA-1	1065	117371.38	5.75	8628	-0.205	Chloroplast
FaPP2C125	maker-Fvb4-2-snap-gene-90.46-mRNA-1	807	88722.82	5.52	7278	-0.200	Chloroplast
FaPP2C135	maker-Fvb4-3-snap-gene-198.55-mRNA-1	1079	118956.91	5.77	9983	-0.244	Chloroplast
FaPP2C134	maker-Fvb4-3-snap-gene-133.59-mRNA-1	1073	118424.41	5.77	8428	-0.234	Chloroplast
FaPP2C158	maker-Fvb6-1-augustus-gene-320.50-mRNA-1	376	41954.43	5.76	3773	-0.367	Nuclear
FaPP2C185	maker-Fvb6-4-augustus-gene-52.28-mRNA-1	376	41873.46	5.76	3427	-0.328	Nuclear
FaPP2C174	maker-Fvb6-3-augustus-gene-377.38-mRNA-1	240	26639.29	6.11	2508	-0.297	Chloroplast
FaPP2C29	augustus_masked-Fvb6-2-processed-gene-234.13-mRNA-1	752	87386.90	6.53	6615	-0.918	Nuclear
FaPP2C175	maker-Fvb6-3-augustus-gene-434.28-mRNA-1	453	50346.30	5.00	2269	-0.519	Cytoplasm
FaPP2C172	maker-Fvb6-3-augustus-gene-354.31-mRNA-1	453	50450.37	5.00	2373	-0.544	Chloroplast
FaPP2C187	maker-Fvb6-4-snap-gene-75.37-mRNA-1	515	57228.39	5.40	9319	-0.466	Nuclear
FaPP2C156	maker-Fvb6-1-augustus-gene-300.38-mRNA-1	453	50273.16	4.96	2699	-0.532	Chloroplast
FaPP2C184	maker-Fvb6-4-augustus-gene-322.34-mRNA-1	471	52126.43	5.21	4435	-0.446	Chloroplast
FaPP2C167	maker-Fvb6-2-augustus-gene-323.28-mRNA-1	473	52419.81	5.28	3533	-0.454	Chloroplast
FaPP2C160	maker-Fvb6-1-augustus-gene-54.55-mRNA-1	473	52333.71	5.47	3414	-0.452	Chloroplast
FaPP2C176	maker-Fvb6-3-augustus-gene-47.28-mRNA-1	473	52428.79	5.35	3828	-0.464	Chloroplast
FaPP2C103	maker-Fvb3-3-augustus-gene-268.37-mRNA-1	534	58808.28	5.00	5575	-0.375	Nuclear
FaPP2C14	augustus_masked-Fvb3-2-processed-gene-288.4-mRNA-1	534	58595.84	5.00	4098	-0.399	Nuclear
FaPP2C12	augustus_masked-Fvb3-1-processed-gene-29.2-mRNA-1	534	58784.16	5.05	4026	-0.420	Nuclear
FaPP2C182	maker-Fvb6-4-augustus-gene-25.60-mRNA-1	496	54655.27	5.39	4207	-0.445	Chloroplast
FaPP2C22	snap_masked-Fvb6-3-	496	54608.39	5.33	5598	-0.417	Chloroplast

	8	processed-gene-427.37-mRNA-1						
	FaPP2C165	maker-Fvb6-2-augustus-gene-269.56-mRNA-1	497	54676.21	5.21	5263	-0.451	Chloroplast
	FaPP2C159	maker-Fvb6-1-augustus-gene-356.68-mRNA-1	497	54618.17	5.26	4869	-0.440	Chloroplast
	FaPP2C130	maker-Fvb4-3-augustus-gene-69.40-mRNA-1	424	45650.97	7.03	5017	-0.113	Cytoplasm
	FaPP2C16	augustus_masked-Fvb4-3-processed-gene-66.7-mRNA-1	424	45660.99	7.56	5227	-0.117	Cytoplasm
	FaPP2C15	augustus_masked-Fvb4-1-processed-gene-92.9-mRNA-1	422	45445.87	8.45	5142	-0.133	Cytoplasm
	FaPP2C18	augustus_masked-Fvb4-4-processed-gene-155.3-mRNA-1	430	46458.91	7.56	6029	-0.137	Cytoplasm
	FaPP2C120	maker-Fvb4-2-augustus-gene-143.22-mRNA-1	434	46718.08	7.04	5121	-0.122	Cytoplasm
	FaPP2C96	maker-Fvb3-2-augustus-gene-157.40-mRNA-1	302	34050.50	8.59	1960	-0.614	Nuclear
	FaPP2C94	maker-Fvb3-1-snap-gene-130.52-mRNA-1	201	21685.40	5.95	1577	-0.203	Chloroplast
	FaPP2C107	maker-Fvb3-4-augustus-gene-155.33-mRNA-1	409	46286.74	5.27	7995	-0.601	Nuclear
	FaPP2C13	augustus_masked-Fvb3-2-processed-gene-157.5-mRNA-1	127	13562.03	4.44	880	-0.176	Cytoplasm
	FaPP2C88	maker-Fvb3-1-augustus-gene-149.29-mRNA-1	312	34952.33	5.04	2141	-0.464	Chloroplast
K	*FaPP2C105*	maker-Fvb3-3-snap-gene-134.38-mRNA-1	406	45763.47	5.62	2314	-0.465	Endoplasmic reticulum
	FaPP2C98	maker-Fvb3-2-augustus-gene-259.23-mRNA-1	397	43796.44	5.39	2692	-0.401	Nuclear
	FaPP2C92	maker-Fvb3-1-augustus-gene-54.22-mRNA-1	397	43687.30	5.48	2133	-0.416	Nuclear
	FaPP2C101	maker-Fvb3-3-augustus-gene-242.27-mRNA-1	397	43544.15	5.38	2342	-0.387	Nuclear
	FaPP2C112	maker-Fvb3-4-snap-gene-58.51-mRNA-1	367	40197.59	5.54	1697	-0.379	Nuclear
	FaPP2C203	maker-Fvb7-3-augustus-gene-69.48-mRNA-1	422	45714.71	5.93	2490	-0.354	Cytoplasm
	FaPP2C190	maker-Fvb7-1-augustus-gene-286.66-mRNA-1	422	45540.45	5.93	2413	-0.355	Cytoplasm

FaPP2C197	maker-Fvb7-2-augustus-gene-289.60-mRNA-1	422	45401.32	6.13	2455	-0.356	Chloroplast
FaPP2C195	maker-Fvb7-2-augustus-gene-231.42-mRNA-1	422	45346.19	5.86	2623	-0.359	Chloroplast
FaPP2C206	maker-Fvb7-4-augustus-gene-54.63-mRNA-1	422	45567.51	5.86	2490	-0.340	Chloroplast
FaPP2C207	maker-Fvb7-4-augustus-gene-61.62-mRNA-1	422	45390.40	6.13	2550	-0.323	Chloroplast
FaPP2C34	augustus_masked-Fvb7-2-processed-gene-286.2-mRNA-1	389	42658.22	5.70	2007	-0.315	Nuclear
FaPP2C202	maker-Fvb7-3-augustus-gene-22.46-mRNA-1	356	39528.83	8.20	1836	-0.381	Nuclear
FaPP2C205	maker-Fvb7-4-augustus-gene-11.33-mRNA-1	387	42714.54	7.58	2246	-0.336	Nuclear
FaPP2C193	maker-Fvb7-1-augustus-gene-312.22-mRNA-1	389	42702.21	5.86	2110	-0.306	Nuclear
FaPP2C191	maker-Fvb7-1-augustus-gene-296.38-mRNA-1	387	42416.90	5.86	2087	-0.288	Nuclear
FaPP2C21	augustus_masked-Fvb5-1-processed-gene-32.19-mRNA-1	350	37351.79	5.15	1053	-0.252	Chloroplast
FaPP2C20	augustus_masked-Fvb5-1-processed-gene-28.2-mRNA-1	350	37466.98	5.25	1053	-0.254	Chloroplast
FaPP2C23	augustus_masked-Fvb5-2-processed-gene-22.5-mRNA-1	348	37285.72	5.23	1047	-0.250	Chloroplast
FaPP2C25	augustus_masked-Fvb5-3-processed-gene-250.14-mRNA-1	276	29581.96	5.55	1042	-0.364	Nuclear
FaPP2C27	augustus_masked-Fvb5-4-processed-gene-25.16-mRNA-1	349	37358.81	5.16	1050	-0.228	Chloroplast
FaPP2C66	maker-Fvb1-4-snap-gene-25.58-mRNA-1	479	52775.10	5.77	4240	-0.129	Chloroplast
FaPP2C42	maker-Fvb1-1-augustus-gene-248.49-mRNA-1	371	40542.64	5.28	4638	-0.289	Chloroplast
FaPP2C48	maker-Fvb1-2-augustus-gene-27.54-mRNA-1	411	45464.25	5.45	4665	-0.196	Chloroplast
FaPP2C01	augustus_masked-Fvb1-3-processed-gene-0.8-mRNA-1	291	31958.37	5.18	1287	-0.212	Chloroplast
FaPP2C02	augustus_masked-Fvb1-3-	144	15591.11	4.83	1299	-0.585	Nuclear

	processed-gene-28.9-mRNA-1						
FaPP2C63	maker-Fvb1-4-augustus-gene-34.51-mRNA-1	471	51614.24	5.31	5267	-0.331	Chloroplast
FaPP2C52	maker-Fvb1-2-augustus-gene-8.49-mRNA-1	473	51715.34	5.32	2693	-0.310	Chloroplast
FaPP2C41	maker-Fvb1-1-augustus-gene-239.61-mRNA-1	473	51727.36	5.37	2952	-0.296	Chloroplast
FaPP2C56	maker-Fvb1-3-augustus-gene-38.41-mRNA-1	475	51949.54	5.31	3292	-0.313	Chloroplast
FaPP2C50	maker-Fvb1-2-augustus-gene-57.60-mRNA-1	473	51745.35	5.32	2710	-0.317	Chloroplast
FaPP2C69	maker-Fvb2-1-augustus-gene-208.33-mRNA-1	548	59356.85	4.63	4443	-0.162	Chloroplast
FaPP2C85	maker-Fvb2-4-augustus-gene-222.40-mRNA-1	548	59318.69	4.59	4439	-0.187	Chloroplast
FaPP2C76	maker-Fvb2-3-augustus-gene-0.25-mRNA-1	547	59173.53	4.59	4464	-0.196	Chloroplast
FaPP2C74	maker-Fvb2-2-augustus-gene-15.47-mRNA-1	548	59331.77	4.59	4445	-0.192	Chloroplast
FaPP2C132	maker-Fvb4-3-augustus-gene-91.56-mRNA-1	1254	140174.78	6.46	5150	-0.217	Golgi apparatus
FaPP2C131	maker-Fvb4-3-augustus-gene-91.47-mRNA-1	534	59803.09	5.81	1897	-0.074	Chloroplast
FaPP2C153	maker-Fvb6-1-augustus-gene-123.54-mRNA-1	552	59373.36	4.90	3715	-0.082	Extracellular matrix
FaPP2C169	maker-Fvb6-3-augustus-gene-118.44-mRNA-1	552	59584.67	4.91	4370	-0.091	Chloroplast
FaPP2C183	maker-Fvb6-4-augustus-gene-255.31-mRNA-1	552	59678.76	4.97	3513	-0.094	Chloroplast
FaPP2C166	maker-Fvb6-2-augustus-gene-27.57-mRNA-1	552	59466.45	4.87	3731	-0.083	Chloroplast
