# Supplementary material for: Genome-Wide Identification and Abiotic Stress Response Analysis of PP2C Gene Family in Woodland and Pineapple Strawberries
Source: Int J Mol Sci. 2023 Feb 17;24(4):4049. doi: 10.3390/ijms24044049 (PMC9961684; doi:10.3390/ijms24044049)
Supplement: Supplementary file 1 [file ijms-24-04049-s001.zip › Supplementary Table S3.pdf]

Supplementary Table S3. Primer sequences of FvPP2C gene family used for qRT-PCR.

Gene	Primer sequence (5'-3')	
FvPP2C01	F: AGGTGTCGGCGATCCAGGAAG	R: AGTTGTCACAGCACGAACGGATG
FvPP2C02	F: CCTCACCTCCGACGACTCCAG	R: ACGGCGTCCTCCATCTCCTTC
FvPP2C03	F: AGGCTGCTGGAGGCAAGGTC	R: TCACTGGCTAGGATTAGGCACTCG
FvPP2C04	F: ATCGCTAACGCTGGTGATTGTCG	R: CAAGAGCACGCGCCACTGATAG
FvPP2C05	F: GGTTCACGTCAGAGCAACAGTCC	R: ACACCAGCAAGGCAACAAGATCC
FvPP2C06	F: CAGTTCTTGGTCGGAGAGCAATGG	R: CTCCTCACCTCCTCCACTCCAAC
FvPP2C07	F: GCTGAACCAGAGTTCCGAAGGATG	R: CCACGGCGAACAAGGCTAACTG
FvPP2C08	F: TGGCGGAACGGTTGTCAACTG	R: ACCACCACATGCGAATCATCAGG
FvPP2C09	F: GTTCGGAGCAGCAGTCCATGTC	R: ACACCAACAAGGCAGCAAGATCC
FvPP2C10	F: GGCACGAGCACTTGGAGATTGG	R: CACACTGGCTGCTCATCACATCC
FvPP2C11	F: AAGCTGGTAGCCGCACATGC	R: GCACCTGATTGATACTCGGACTCC
FvPP2C12	F: TGTTGGAAGGTTGTTGGTGGTAGC	R: ACAGATCCTCCACATGCCTCGAC
FvPP2C13	F: GAGTTCCACTGAGGCGGTTGC	R: CCAGCCATCACGTTACCAGTAGC
FvPP2C14	F: AGCTCCTTCCAGGTCTAGCCATG	R: TGGAGGTCAGGCGACGATGG
FvPP2C15	F: CCGTTGGTGATTCTCGTTGCATTG	R: CCGCCGCCAGTATTAAGCCTAC
FvPP2C16	F: GCATGAACAGCTAAGCTCGGAGAG	R: TGGTGCTTCGGTTGTTGAGGATTC
FvPP2C17	F: GGTCAACTGCTTCCACTGCTATCC	R: CCTCAATCCGTTGCCGCTCATC
FvPP2C18	F: TGGTGACCTGAAGGAGCACA	R: TTCCACAAACCATCACTTGCCA
FvPP2C19	F: ACAGAGGATGCAGTCAGGAGAGC	R: AGCTGCTTAGCCACACCATTCTTG
FvPP2C20	F: GGCGTTCGGAGAGCATTACTGATG	R: CGCAGTTCCTCCACCACGAATTG
FvPP2C21	F: TCCACTCGGAAGAAGCCACCAC	R: AGCGGAATCGAGGCGAAGGAG
FvPP2C22	F: CTTCTTCTGCCAGCAGGACCAAG	R: ACACTTCGTCGCCACATGAGAAC
FvPP2C23	F: CCTCGCAGCAATCTTCGTCTTCG	R: GGTCCAGCAACTGAAGCCGATAAC
FvPP2C24	F: GACTGTGCCGTTAGAGCTTGGAG	R: CTCTGTCACAACCTCATCCGCTAC
FvPP2C25	F: AACCTTCATGGATGATGCCAGCAG	R: ACAGATGTGCCTGCATGTACTTGG
FvPP2C26	F: CTTACCGTCGCCTCACTGACAAG	R: AACGGCACAGTCATCAGTCTTGG
FvPP2C27	F: CCGGAAGTGTCAGTGGTTCAGC	R: TCTCCAGCAATGGCAGCACATG
FvPP2C28	F: CAATGCAATGGTGACAGAGTTGGC	R: GGCACTTAACGCTGCTCCAGAC
FvPP2C29	F: CAATGCAATGGTGACAGAGTTGGC	R: GGCACTTAACGCTGCTCCAGAC
FvPP2C30	F: TTCAAGCCTAACTTGCCTCAGGAG	R: GATACTGCCAGTCCAGGTGTCTTC
FvPP2C31	F: TAGCGACTGTTGGAACATGCTGTC	R: TCGCATTGTGTTCGGTGGATAGC
FvPP2C32	F: ACTTGGCTCCTCGTCACCTCTC	R: GCTCACGCCTTACAAGACTCACC
FvPP2C33	F: CGTTAGTCGATGAGCAGGAGGTTG	R: TGCCGCAGTATGATCCGTTGTAAG
FvPP2C34	F: GGAACAGTCAGCAGAGGCATCAC	R: ACGGCTCTTGAATCACCACAGTTC
FvPP2C35	F: TTGCCTGCTGAACTTGCTGCTAG	R: TGGAGGCTGTACCGAGTTATCAGG
FvPP2C36	F: GTGCTATCTACGTCACGCTCGATC	R: GTCCGTCGCTCGCTATCAACAG
FvPP2C37	F: GGAGCGTGCTCGTATTCAGAAGG	R: CAGAGGCCGTCGCAACCAAG
FvPP2C38	F: GCGTCCGTCAGTGCCAACAG	R: AAGCCGCCGAGGACACTCTC
FvPP2C39	F: TGGCTCGTGCTCTAGGAGATTGG	R: TCTTCCTCGGTCAGGCATGTCTC
FvPP2C40	F: GACCAGGCAATCCTCTCACATAGC	R: ATGCTGCCTCGTTCAGTGTTGG
FvPP2C41	F: TCCTGCTTGTCCGCCGAGAG	R: GCAGCTTGAGTAGAGCCATTGAGG

FvPP2C42	F: TCCGAGCCTGAAGTCACTCACAG	R: CGACGAGTCGCTTGGCACAC
FvPP2C43	F: TGGTCGTGAAGGAGGCTCTGAG	R: GGCAAGACAGGACGTTCTGATGG
FvPP2C44	F: GTCGGTCAATGTCTCATGGAAGCC	R: ACAGTTCGGTGCCTCCTCCATC
FvPP2C45	F: GTGTTCCGGTCCGATTGAGAAGC	R: CCGACTTGTGCCGCCGATTC
FvPP2C46	F: CATCTTAGCAACCAGGAGGCTGTC	R: TGTCGTCTCACTCCTCGCTCTATC
FvPP2C47	F: AGCCAATGCGGTTCTTGAGGATC	R: GCGGCATCAGGTCCACCATG
FvPP2C48	F: GGATGGATGAGATGATGCGTGGAC	R: TTCTCTTGGCTTCAGCACTCTTGG
FvPP2C49	F: CAGAGGCATCAGCGGAAGTAACG	R: TGGCTCTTGAATCACCGCAGTTC
FvPP2C50	F: CAGAGCGAGACCAGTTCATTGTCC	R: TGAGCGGCTTGGAGCTGAGG
FvPP2C51	F: GCCGTGTTGGAGACCTTCTGTTG	R: AAGCAAGCTCAACGGTCCAGATTC
FvPP2C52	F: CGTCTCCAATTGCGGCGACTC	R: ATGGCGAGCACTCCGAGGAC
FvPP2C53	F: TGGTGTCGAATGCTGGTGATTGTC	R: GCTGGCGATCTCCAATTCCTCTTG
FvPP2C54	F: ATTGCTGGTGCTTGCTAGTGATGG	R: GCTGCTGCCTCAGGTTCTTCTTC
FvPP2C55	F: TGACGAACCGAACAGACAAGGATG	R: CAGGAGTACACGATCACGATCACG
FvPP2C56	F: AAGAGATGGAAGACGGCGTTGTTG	R: CTTGACAGAGGAGAAGCCAGCATG
FvGAPDH	F: CATTCATCACCACCGACTACA	R: GAAGGGTCTTCTCATCCTTGAC
