# Supplementary figures and images for: Genome-Wide Identification and Abiotic Stress Response Analysis of PP2C Gene Family in Woodland and Pineapple Strawberries
Source: Int J Mol Sci. 2023 Feb 17;24(4):4049. doi: 10.3390/ijms24044049 (PMC9961684; doi:10.3390/ijms24044049)

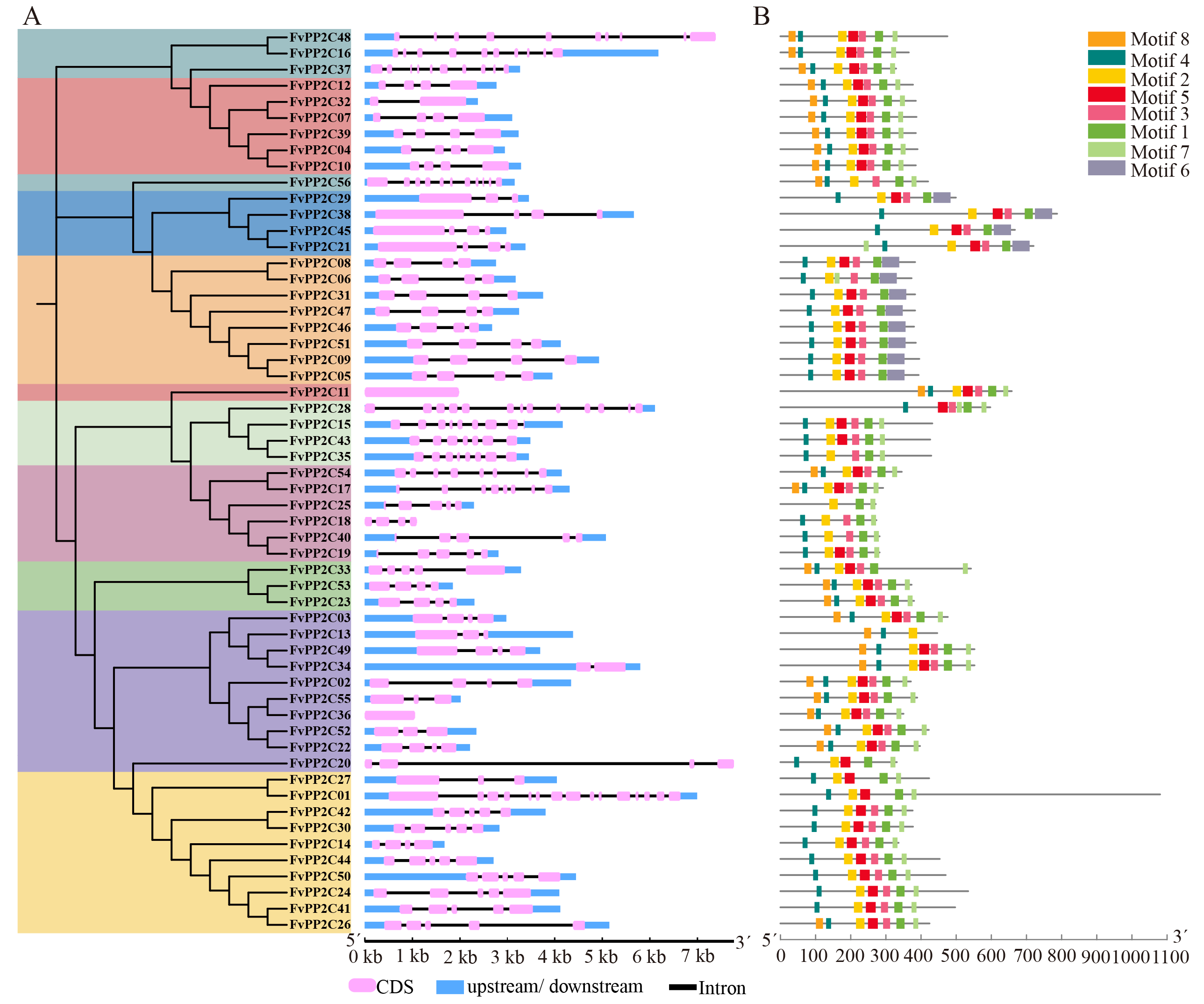

Supplement: Supplementary file 1 [file ijms-24-04049-s001.zip › Supplementary Figure S1.tif]

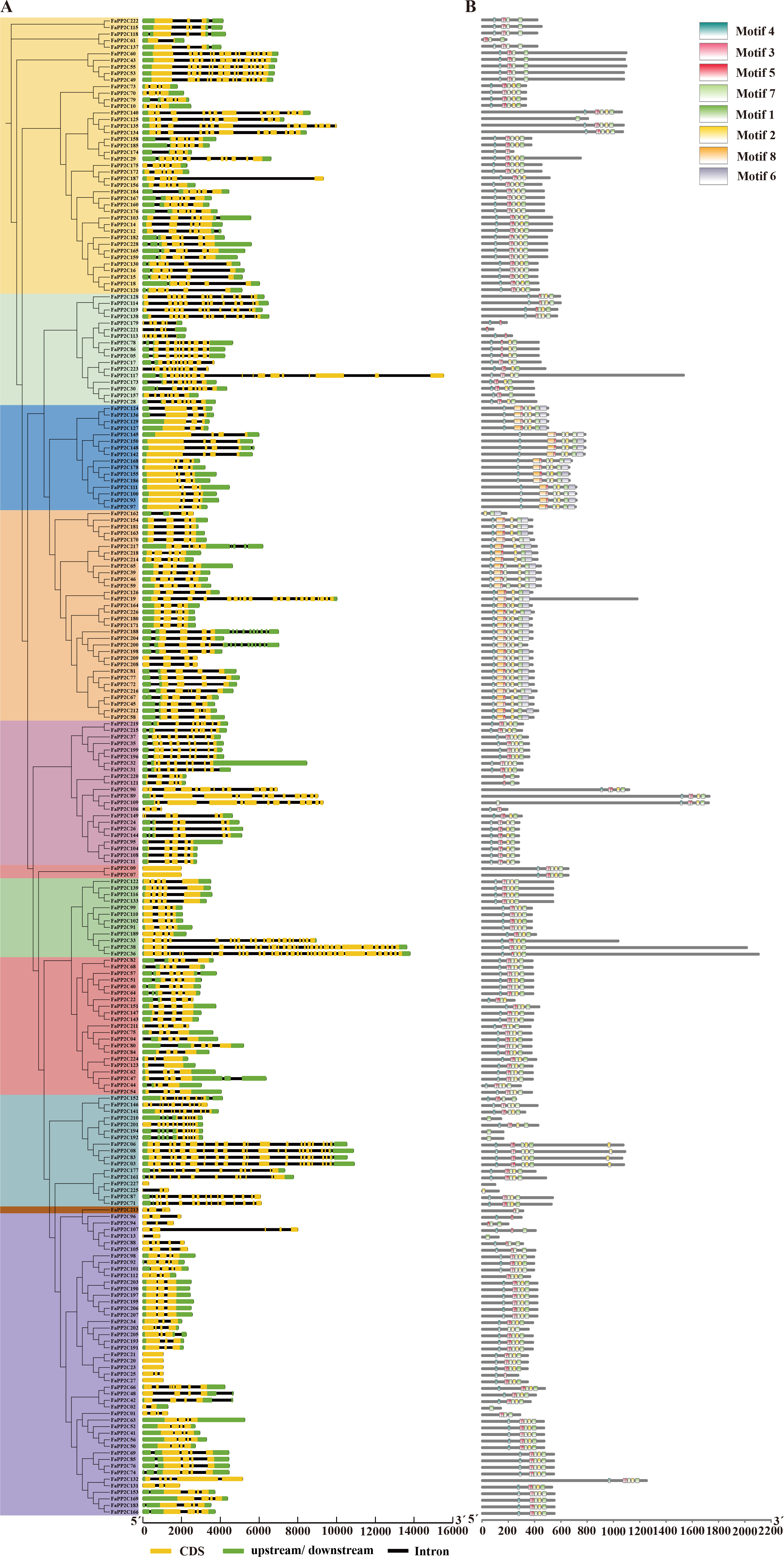

Supplement: Supplementary file 1 [file ijms-24-04049-s001.zip › Supplementary Figure S2.tif]

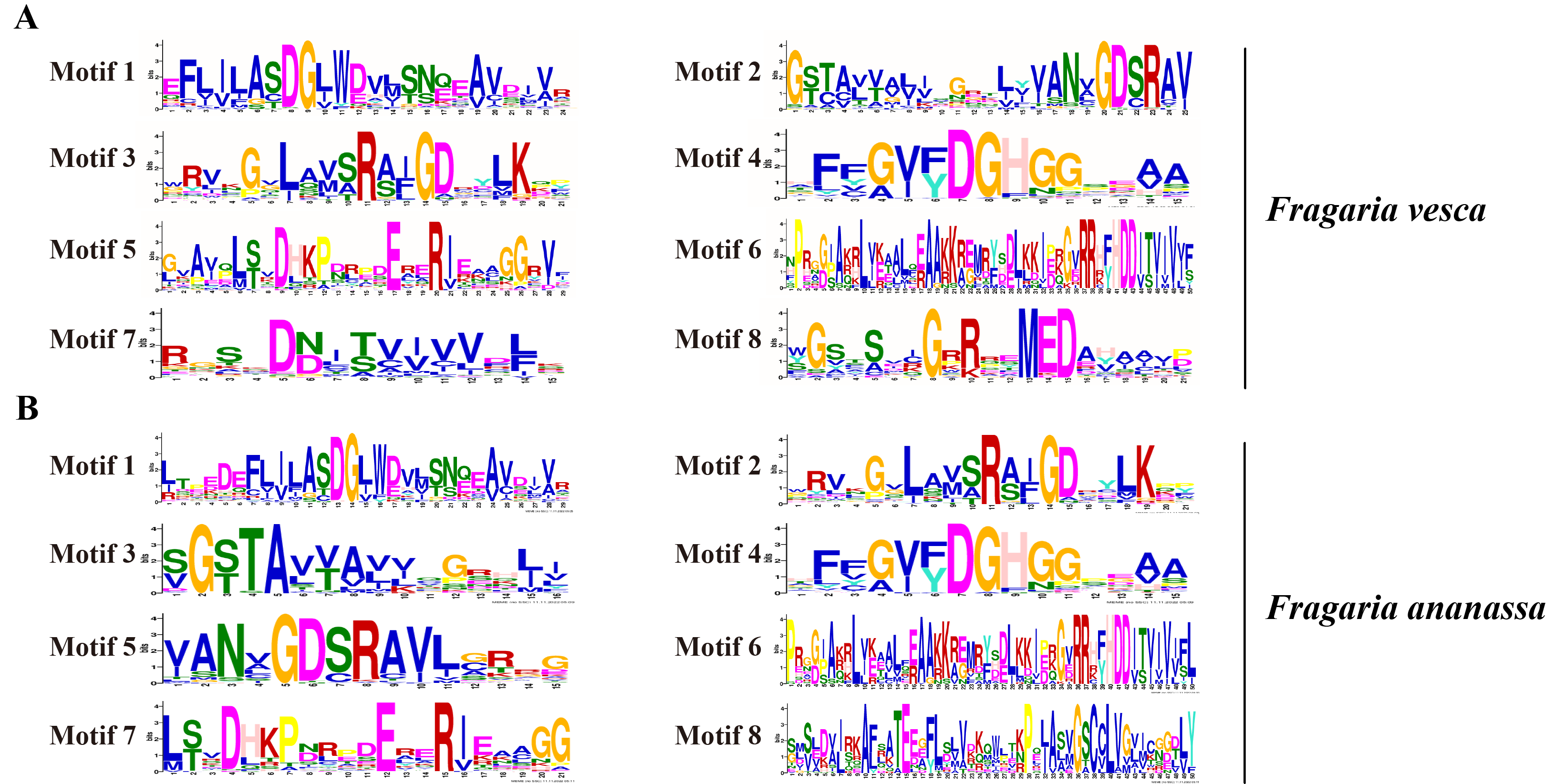

Supplement: Supplementary file 1 [file ijms-24-04049-s001.zip › Supplementary Figure S3.tif]
